# Barriers and facilitators to sexual and reproductive healthcare access for women with severe mental illness in low- and middle-income countries: A qualitative systematic review and meta-aggregation

**DOI:** 10.1017/gmh.2026.10222

**Published:** 2026-05-20

**Authors:** Anushka Chalmeti, Bhuvan Prathap, Simran Samudre, Carrie Brooke-Sumner, Charlotte Hanlon, Ribka Birhanu, Laura Asher

**Affiliations:** 1 Columbia University Mailman School of Public Health, USA; 2 University of Nottingham School of Medicine, UK; 3 Alcohol Tobacco and Other Drug Research Unit, South African Medical Research Council, South Africa; 4 University of Edinburgh, UK; 5 Addis Ababa University College of Health Sciences, Ethiopia; 6 Unit of Lifespan and Population Health, University of Nottingham School of Medicine, UK

**Keywords:** severe mental illness, women’s health, reproductive health, sexual health, low- and middle-income countries

## Abstract

Women with severe mental illnesses (SMI) in low- and middle-income countries (LMICs) appear to have poorer sexual and reproductive health (SRH) compared to women without SMI and men with SMI. Previous research suggests that women with SMI experience higher rates of HIV and other sexually transmitted diseases, sexual violence and variable contraceptive use. This review seeks to identify key barriers and facilitators affecting access to SRH services for women with SMI in LMICs by systematically synthesizing relevant research. A qualitative systematic review was conducted using the Joanna Briggs Institute (JBI) methodology. Electronic searches were made on July 31, 2024, in MEDLINE, EMBASE, PsycINFO and citation tracking covering the period from 2004 to 2024. Studies conducted in LMICs that explored barriers and facilitators for accessing SRH services for women aged 18 years or older diagnosed with SMI were included. Data extraction and methodological quality assessments were completed and findings were compiled using the JBI meta-aggregation approach. The confidence of these findings was evaluated using the ConQual method. Eleven studies met the inclusion criteria. Four synthesized findings were: (a) Variable knowledge of SRH and low risk perception hinder access to SRH care for women with SMI; (b) Service availability shapes access to SRH care for women with SMI; (c) Restricted autonomy and exclusion from health decisions prevent women with SMI from accessing SRH care that reflects their needs and rights; and (d) Stigma and gendered expectations around mental illness and sexuality, reinforced by societal norms, discourage care-seeking for the SRH needs of women with SMI. All of these findings were assessed to have moderate confidence. Women with SMI in LMICs face distinct challenges in accessing SRH services. By considering the multifaceted barriers and facilitators identified in this review, policymakers can create more targeted and equitable interventions that improve accessibility of SRH services for this population.

## Impact statements

This review shares findings on a historically overlooked public health crisis: access to sexual and reproductive health (SRH) care for women with severe mental illness (SMI) in low- and middle-income countries (LMICs). By synthesizing qualitative evidence from the past two decades, this study identifies barriers and facilitators that hinder or promote access to SRH care. Our findings show how women with SMI face barriers, such as sociocultural, autonomy-related, and systemic inaccessibility challenges that impact access to SRH care. This has broad implications for mental health integration in global systems and reproductive and gender equity, especially for women with SMI – a group that faces intersectional, layered barriers to care due to sexism, ableism, stigma and institutional marginalization. This research can contribute to evidence-based interventions for international organizations, humanitarian groups and local policymakers to create targeted, community-specific interventions that address the nuances of access to both reproductive and mental health services for women in LMICs. By amplifying the experiences of women with SMI, their caregivers, and healthcare providers, this study aims to further research that assesses inclusive SRH care for women with SMI and advocates for a global health agenda that is more inclusive, comprehensive and rights-based.

## Introduction

Sexual and reproductive health (SRH) care encompasses access to effective contraception, evidence-based sexual education, appropriate sexually transmitted infection (STI) treatment, safe antenatal, childbirth and postnatal care, and the right to medical treatment and legal action in case of sexual violence. Accessing SRH care is fundamental to the realization of SRH and rights, yet millions of women worldwide, especially those in low- and middle-income countries (LMICs), remain chronically underserved (Starrs et al., 2018; Kaul et al., 2024). Five dimensions of healthcare accessibility have been identified, and these can be applied to SRH care: (a) approachability: the ability to perceive a need for care; (b) acceptability: the ability to seek care within the constraints of sociocultural values and expectations; (c) availability and accommodation: the ability to reach care, both physically and in a timely manner; (d) affordability: the ability to pay for care through income or social capital; and (e) appropriateness: the ability to engage with care in a way that is adequate for an individual’s unique needs (Levesque et al., 2013). This five-pronged framework can help distinguish facilitators of care access and evaluate the alignment between healthcare systems and the needs of individuals, families and communities, allowing the conception of more comprehensive interventions (McIntyre et al., 2009). Despite global commitments to bettering access to SRH care, such as Sustainable Development Goal 3.7, which aims for universal access to SRH services by 2030, social and institutional barriers, like shame, stigma and lack of provision of care, continue to prevent women in LMIC from accessing appropriate and necessary SRH care. The effects of poverty, sociocultural pressures and systemic underfunding of, and widespread stigma surrounding, SRH services restrict the accessibility of SRH care in these settings (Desrosiers et al., 2020).

Among the most marginalized are women living with severe mental illness (SMI), which are mental illnesses associated with enduring disability, including severe depression, schizophrenia and bipolar disorder. The United Nations Convention on the Rights of Persons with Disabilities (CRPD) states that people with disabilities, which includes people living with SMI, should have access to the same range and quality of affordable SRH care as provided to other persons (United Nations, 2006). Yet due to the intersectional layers of gender and health status discrimination, women with SMI face compounded barriers to SRH care that reflect not only stigma around mental illness, but also sexism, ableism and institutional marginalization; this can manifest through limited access to menstrual products and contraception, SRH education and abortion services (Howard et al., 2025). In under-served populations, these challenges can be intensified: for instance, in LMIC settings, women with SMI are vulnerable to heightened risks of HIV and other STIs, sexual dysfunction and stigma, contraception avoidance or coercion, sexual violence and complexities in pregnancy (Sisodia et al., 2025).

Evidence synthesis is crucial because while empirical research usually focuses on women with single diagnoses or in specific countries, a broader synthesized understanding is necessary to identify predominant themes, recurring gaps and contextual variations in access to care. A previous scoping review identified a number of qualitative studies exploring the SRH needs of women with SMI in LMICs but did not include in-depth analysis (Sisodia et al., 2025). A previous systematic review examined barriers and facilitators in accessing SRH care for women with SMI, but concentrated primarily on HIV and family planning services and drew largely on studies from high-income settings (Brown et al., 2025). While experiences in HIC may have broad relevance (Usman et al., 2025), a focus on LMIC allows us to consider the specific issues that may arise from lower availability of formal resources, more pronounced gender inequalities and diverse cultural and social contexts.

By drawing on qualitative studies that center on lived experiences in various contexts, this qualitative systematic review aimed to address these gaps by exploring the barriers and facilitators to access to SRH care for women with SMI in LMICs.

## Methods

This systematic review was conducted in accordance with the Joanna Briggs Institute (JBI) meta-aggregative approach to the synthesis of qualitative evidence (Munn et al., 2014; Lockwood et al., 2015; Munn et al., 2021). This approach seeks to observe the original meanings reported by the primary researchers. Analysis involves organizing primary study findings into categories and then into synthesized findings that aim to generate meaningful insights and recommendations to inform practice. The review is reported in accordance with the Preferred Reporting Items for Systematic Reviews and Meta-analysis (PRISMA) guidelines (PRISMA, 2020) (see Supplementary File S1). The study protocol was registered with PROSPERO (ID:573143).

### Inclusion and exclusion criteria

We included studies that met the following criteria:

- *Concept*: Studies that explored barriers and facilitators for women with SMI (including schizophrenia, bipolar disorder, severe depression and psychosis) in accessing SRH care (including, but not limited to, care related to family planning, contraception, antenatal and postnatal care, STI treatment, abortion and fertility).
- *Population:* Studies that included women aged 18 years or older diagnosed with an SMI; or included both sexes aged 18 years or older if the results could be differentiated by gender or, in the case of aggregated data, if there were at least 80% women; or included families, community members and professionals that interact with women aged 18 years or older diagnosed with an SMI. We included studies that involved participants with depression, where it was defined by authors as SMI and/or where participants were receiving treatment from specialized facilities.
- *Context:* We included studies conducted in LMICs as defined by the World Bank (World Bank, 2024).
- *Study design*: Peer-reviewed qualitative studies were included with no restriction on study type.

We excluded studies focused only on men and studies focused on women diagnosed with mild depression, post-partum depression, or anxiety disorders.

### Search strategy and study selection

A preliminary search was conducted on MEDLINE using the three concepts: “severe mental illness,” “sexual and reproductive health,” and “low- and middle-income countries.” The final search strategy, developed with a research librarian, combined Medical Subject Headings (MeSH) and keywords (see Supplementary File S2). We searched the electronic bibliographic databases MEDLINE, EMBASE and PsycINFO on July 31, 2024, adapting the strategy as required. The search was limited to studies from 2004 to 2024 to ensure relevance of findings and recommendations to current health systems. Only English language articles were included for logistical reasons (Hannes et al., 2017). The reference lists of included studies were screened for additional studies.

#### Study selection

Studies were imported into Covidence, duplicates removed, and titles and abstracts screened, with 30% double screened due to operational reasons. Any disagreements were resolved through discussion between reviewers. Full texts of relevant studies were then retrieved and screened against inclusion criteria by one reviewer, again due to operational reasons.

#### Assessment of methodological quality

We assessed the methodological quality of eligible studies using the ten criteria of the JBI critical appraisal checklist for qualitative research. Studies were not excluded based on scores because low-quality studies can also generate relevant and usable findings (Lockwood et al., 2015).

#### Data extraction and assessment

We extracted study characteristics (i.e., country, phenomena of interest, participant characteristics, sample size, data collection and analysis methods) from each article using a data extraction tool. Specific findings and respective supporting evidence (i.e., quotations) were extracted and classified as facilitators or barriers. The extracted findings were evaluated for credibility using the following definitions (JBI, 2025):

- **Unequivocal (U):** The findings are accompanied by a contextually rooted, detailed, rich and clearly associated illustration (e.g., participant quote or fieldwork observation).
- **Credible (C):** The findings are accompanied by an illustration lacking detail, richness or clear association with it.
- **Not Supported (NS):** The findings are not supported by the data or there are no data to support the finding.

#### Data synthesis

The findings were grouped into categories and unsupported findings were eliminated to improve the validity and credibility (Lockwood et al., 2015). Categorized findings were then further grouped into synthesized findings. We graded the final synthesized findings using the ConQual approach for establishing confidence in the output of qualitative synthesis. Studies were initially ranked as “high” in terms of both dependability and credibility. We then scored each study for dependability based on five pre-determined questions from the JBI critical appraisal tool, with studies downgraded in the case of low-quality ratings. The overall dependability of each synthesized finding was determined by JBI critical scores (Munn et al., 2014; Munn et al., 2021).

## Results

### Study inclusion

**Figure 1.**
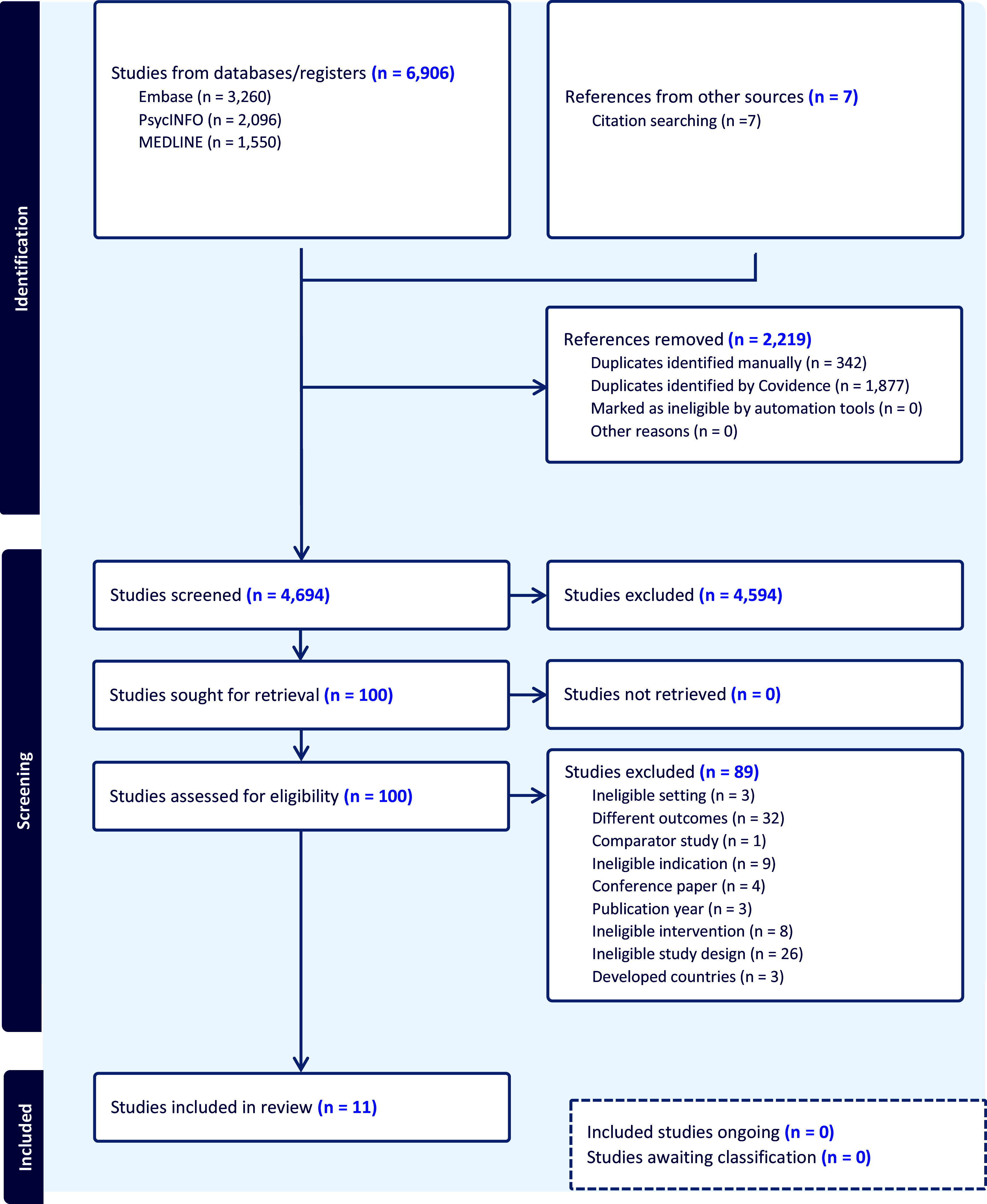
PRISMA flow diagram. Figure 1. long description.

The search identified 6,913 records (Figure 1). Following the removal of 2,219 duplicates, 4,694 publications were screened for inclusion based on the title and abstract. A total of 4,594 studies were excluded at this stage, leaving 100 full texts which were screened for inclusion. Eleven papers met the inclusion criteria.

### Methodological quality

The included studies’ quality ranged from 40%–100%. All the studies met JBI criteria Q3 (congruity between the research methodology and data collection methods), Q4 (congruity between the research methodology and data representation/analysis) and Q7 (influence of the researcher on the research is addressed). The fewest number of studies met Q6 (locating the researcher culturally or theoretically; see Supplementary File S3).

### Characteristics summary of studies included

The included studies were conducted in India (*n* = 4), Uganda (*n* = 2), Iran (*n* = 2), Ethiopia (*n* = 1), Brazil (*n* = 1) and China (*n* = 1) (Table 1). Data collection methods included in-depth, semi-structured or key informant interviews, focus group discussions and participant observation. Data analysis and research methods included thematic analysis, content analysis, and grounded theory. Study participants included women with SMI, family members and healthcare providers.

**Table 1.** Characteristics of the included studies Table 1. long description.

Paper	Country; Setting	Study design	Phenomena of interest	Participant characteristics	Sample size and sampling methods	Data collection and analytical method	Reported limitations
(Bagadia et al., 2020)	India; perinatal psychiatric service	Qualitative study	Determine factors influencing decision-making for pregnancy in women with SMI	Women with SMI: aged 22–36 years	*n* = 42, purposive sampling	Semi-structured interviews; grounded theory approach	Sample from a perinatal psychiatric service, so cannot be generalized to all populations and healthcare settings
(Lundberg et al., 2012)	Uganda; psychiatric inpatient and outpatient services in specialized hospital	Qualitative study	Explore how SMI may influence sexual risk behaviors & sexual abuse	People with SMI: aged 18–49 years	*n* = 20, purposive sampling	Semi-structured interviews; content analysis	Participants with SMI reported to show passivity and reduced speech, reducing data richness
(Raisi et al., 2018)	Iran; psychiatric hospital	Qualitative study	Explore the SRH needs of Iranian patients with mental illness	People with SMI, patients’ family and healthcare professionals: 18 years and older	*n* = 17, purposive sampling	In-depth semi-structured interviews; content analysis	Important issues may not have been fully disclosed by participants
(Rani et al., 2023)	India; tertiary care mental health institution	Qualitative study	Explore the views of people with SMI, caregivers and healthcare professionals on different forms of abuse experienced by people with SMI	People with SMI, experts, caregivers: 18 years and older	*n* = 51, purposive sampling	Focus group discussions; thematic analysis	Study could not capture experiences and perceptions of all the ethnically diverse population, so it cannot be generalized
(Rezaie and Phillips, 2020)	Iran; psychiatric inpatient unit	Qualitative study	Examine the post-discharge needs of women with SMI	Women with SMI: 40 years and older	*n* = 42, purposive sampling	Focus group interviews, conventional content analysis	Only patient’s point of view (did not interview healthcare providers); women with SMI who were not hospitalized were not included
(Tumwakire et al., 2022)	Uganda; mental referral hospital	Qualitative study	Explore mental healthcare workers’ perspective on and experiences with SRH needs of people with SMI	Mental health workers: 18 years and older	*n* = 14, purposive sampling	Semi-structured interviews, in-depth interviews, inductive thematic content analysis	Study conducted at only one site
(Vijayalakshmi et al., 2024a)	India; psychiatric outpatient clinic	Qualitative study	Explore the SRH needs of women with SMI in psychiatric outpatient clinic	Women of reproductive age with SMI: 18–49 years	*n* = 32, purposive sampling	In-depth face-to-face interview, Hycner’s explication for data analysis	Limited to women with sexual dysfunction who were experiencing symptoms of STIs in single setting
(Vijayalakshmi et al., 2024b)	India; tertiary care psychiatric hospitals	Integrated mixed-method study	Understand the SRH needs of women with SMI in tertiary care psychiatric hospitals	Women with SMI: 18–49 years	*n* = 32, purposive sampling	Semi-structured interviews, Hycner’s explication process	Limited generalizability due to selected setting; sexual dysfunction and psychiatric medications not explored
(Wainberg et al., 2007)	Brazil; psychiatric institutions	Ethnography study	Investigate application of existing interventions that are developed in the United States	Patients with SMI: 18–65 years	*n* = 45, purposive sampling	Field observations, focus groups, key-informant interviews and interviews; grounded theory	Limited relevance of SRH interventions that are developed in the United States
(Yu et al., 2022)	China; psychiatric hospital	Descriptive phenomenology study design	Explore the experiences with and perceptions of SRH needs of women with schizophrenia	Women with schizophrenia: 26–40 years	*n* = 15, purposive sampling	Semi-structured interviews, in-depth interviews, Colaizzi’s descriptive analysis framework	Women without desire for children not included
(Zerihun et al., 2021)	Ethiopia; women with SMI who were participating in ongoing population- based cohort study	Qualitative study	Explore the unmet family planning needs of women with SMI	Women with SMI: age 18–49 years	*n* = 16, purposive sampling	In-depth interviews; inductive thematic analysis	Participants recruited from a community-based cohort study and had better access to mental services than general population, so may not be generalizable

### Review findings

**Figure 2.**
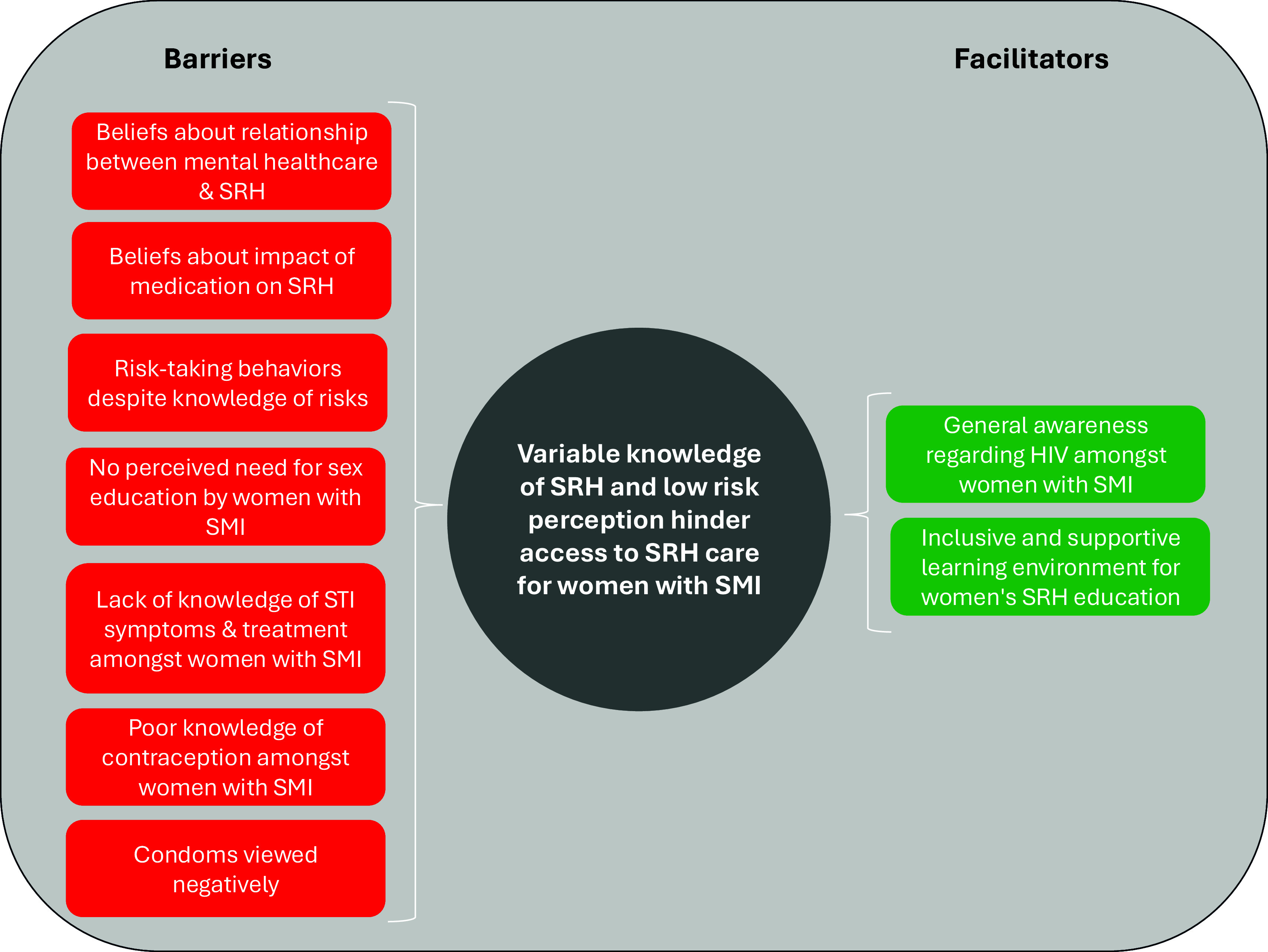
Synthesized finding 1. Figure 2. long description.

**Figure 3.**
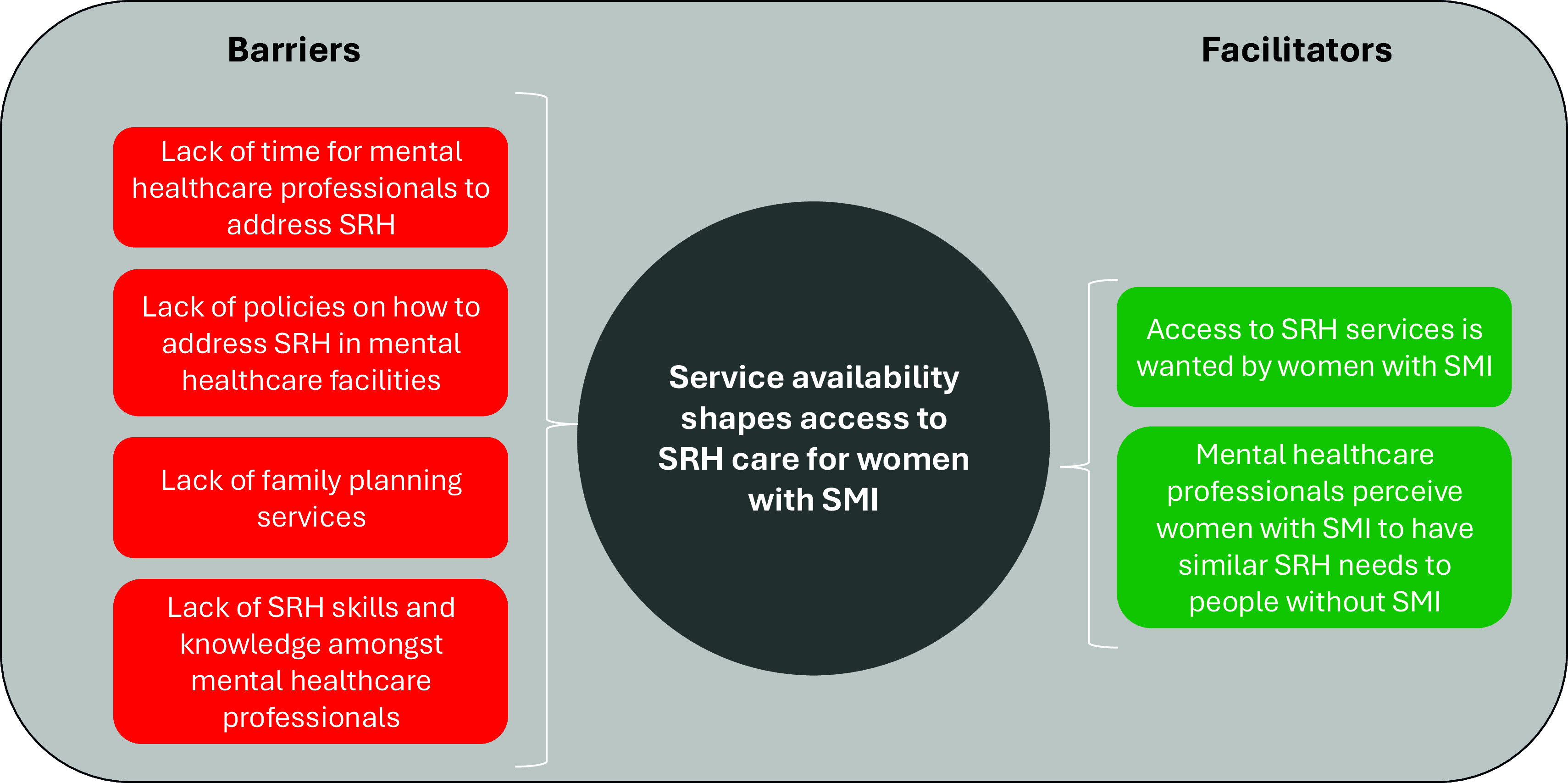
Synthesized finding 2. Figure 3. long description.

**Figure 4.**
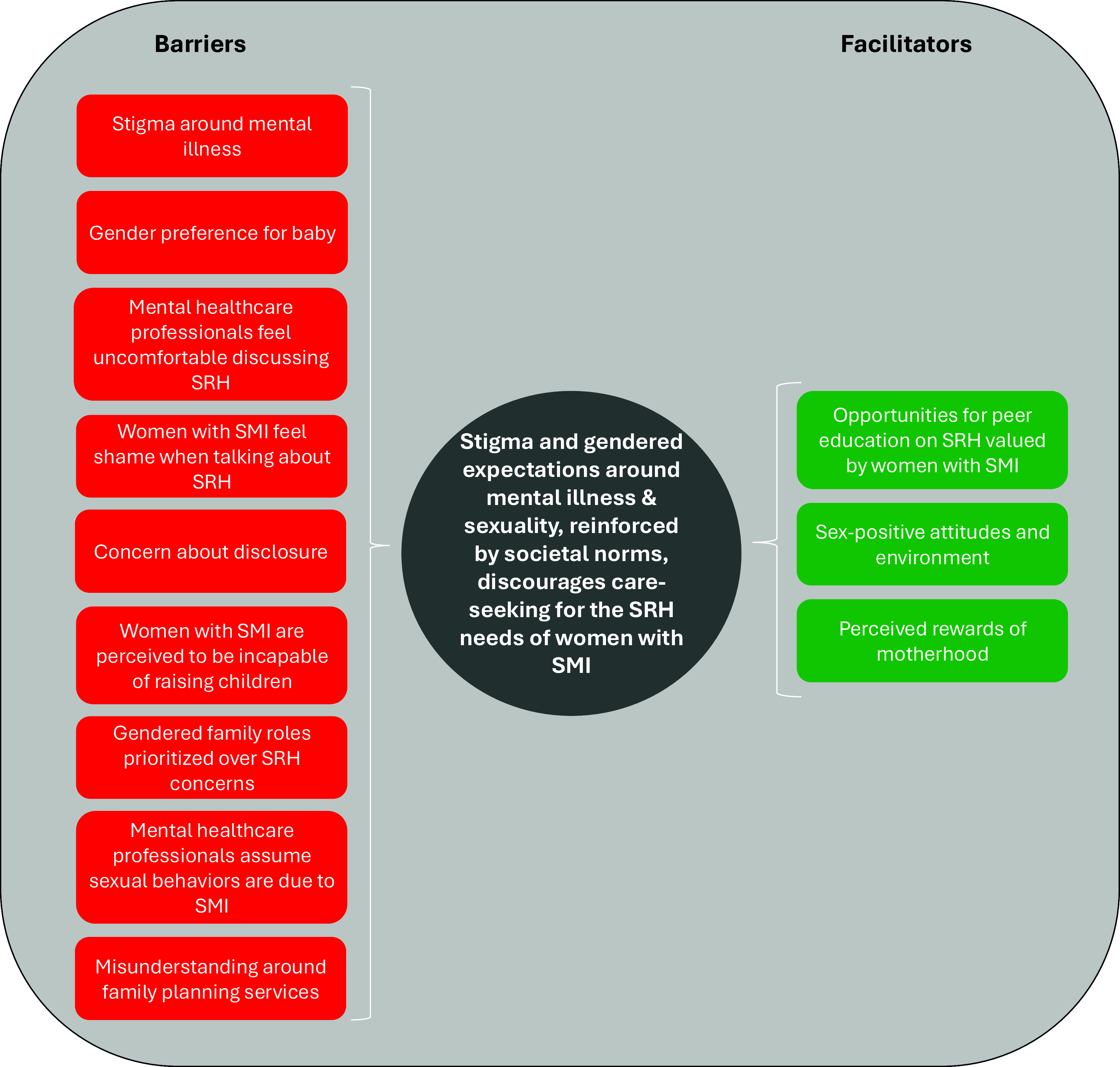
Synthesized finding 3. Figure 4. long description.

**Figure 5.**
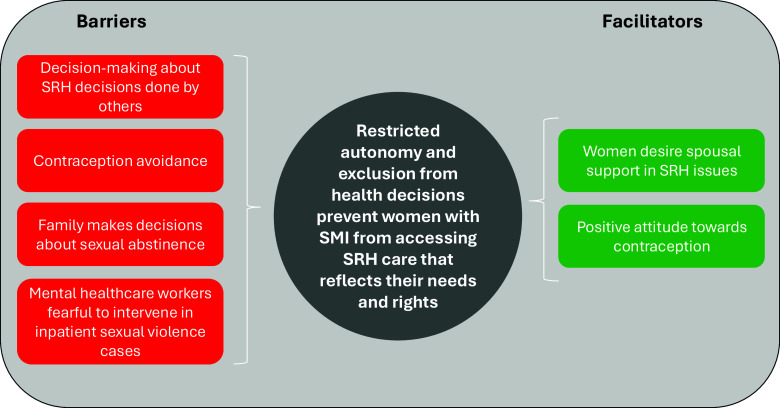
Synthesized finding 4.

From the included studies, a total of 78 findings were extracted. Of these, 74 (~95%) findings were “unequivocal” and 4 (~5%) were “credible.” There were no unsupported findings. Findings were grouped into 33 categories (24 barriers and 9 facilitators), and finally into four synthesized findings (see Supplementary File S4). In Figures 2 to 5, barriers are in red boxes and facilitators are in green boxes with the synthesized finding in the middle.

#### Synthesized finding 1: Variable knowledge of SRH and low risk perception hinder access to SRH care for women with SMI (Figure 2)

##### Barriers

Findings show that women with SMI had variable knowledge about the effects of SMI on their SRH (Yu et al., 2022). Some women were fearful about the effect of SMI on pregnancy, with women in both Iran and India expressing a desire to have a “healthy” baby (Bagadia et al., 2020; Rezaie and Phillips, 2020). Concerns extended to psychiatric treatment for SMI as well. Some women perceived their STI symptoms to be side effects of their psychiatric medication (Vijayalakshmi et al., 2024b), while others feared that their medication would have negative impacts on their fetus (Yu et al., 2022). In response, some women stopped taking psychiatric medication during pregnancy, but subsequently feared relapse of their SMI (Yu et al., 2022). Women with SMI also attributed the experienced decrease in sexual desire to psychiatric medication (Bagadia et al., 2020; Vijayalakshmi et al., 2024b). Despite consulting perinatal psychiatrists, women in an Indian study reported that such views about the effects of medications on SRH remained unchanged (Bagadia et al., 2020), suggesting a gap not in awareness of potential side effects (e.g., reduced sexual desire) but in receiving adequate information about which medications are most likely to cause these effects and what alternative treatment options may exist.

Lack of medical knowledge also manifested in decreased awareness of STI symptoms and treatment among women with SMI (Vijayalakshmi et al., 2024a). Some believed that STI symptoms were a result of other unrelated health conditions, like partners’ smoking addictions (Vijayalakshmi et al., 2024b). When experiencing STI symptoms, some women did not know of the possible treatment options (Vijayalakshmi et al., 2024b).

Low levels of knowledge of contraceptive methods among women with SMI were also reported (Vijayalakshmi et al., 2024a). In women with SMI who were aware of contraceptive methods, negative attitudes toward condoms were common, including beliefs that condoms were linked to promiscuity (Zerihun et al., 2021). These attitudes were apparent in healthcare professionals too, with one stating that access to condoms for women with SMI in psychiatric facilities will encourage sexual activity. Even among women with SMI who were knowledgeable about STIs, they did not report practicing safer sex; mental healthcare professionals confirmed this, saying that despite the availability of condoms, they were not used by people with SMI (Wainberg et al., 2007). Women in this study reported that Brazilian churches have claimed that condoms are not efficacious as an HIV transmission prevention method, rather encouraging worshippers to believe in religious protective mechanisms (Wainberg et al., 2007).

Despite these numerous challenges, some women also expressed that they did not need sexual health education (Raisi et al., 2018).

##### Facilitators

Other women with SMI did report a need for sexual education concerning SMI and sexual relations to maintain normal relations with their partners (Rezaie and Phillips, 2020). Some women reported already having a baseline knowledge of HIV symptoms from governmental health campaigns (Wainberg et al., 2007). There was also a call for inclusive learning environments for SRH education, especially ones that are gender-inclusive, culturally sensitive and foster community (Wainberg et al., 2007).

#### Synthesized finding 2: Service availability shapes access to SRH care for women with SMI (Figure 3)

##### Barriers

Many mental healthcare providers expressed an inability to address SRH concerns due to a lack of time. Some admitted to not addressing SRH at all, unless the issue is raised specifically by the patient (Tumwakire et al., 2022). Others believed it was a better use of time to treat SMI symptoms than it was to address SRH, especially given short inpatient stays (Raisi et al., 2018). Another factor was insufficient training: some providers believed that they did not have adequate education or the appropriate medical specialization (Raisi et al., 2018). Women participants mirrored this sentiment, saying that they did not think their mental healthcare professionals were equipped to handle SRH concerns. Mental healthcare professionals also felt there were no policies and governance to address SRH needs and behaviors (Tumwakire et al., 2022). One mental healthcare professional expressed frustration with the lack of proper hospital protocol and training for addressing sexual interactions between patients in psychiatric facilities (Wainberg et al., 2007). Women in the only community-based study reported a lack of family planning services (Zerihun et al., 2021).

##### Facilitators

In some studies, women wanted increased access to SRH services (Rezaie and Phillips, 2020; Zerihun et al., 2021). Women with SMI highlighted a need for advice on pregnancy-related topics and some suggested that family planning services be integrated into mental healthcare (Zerihun et al., 2021). Health professionals supported this need for family planning (Tumwakire et al., 2022). Many mental healthcare professionals perceived women with SMI to have similar SRH needs to people without SMI. They also believed that intimate relationship maintenance and security were big motivators for continuing with psychiatric treatment (Tumwakire et al., 2022).

#### Synthesized finding 3: Restricted autonomy and exclusion from health decisions prevent women with SMI from accessing SRH care that reflects their needs and rights (Figure 4)

##### Barriers

Women with SMI across diverse contexts described decreased autonomy in making decisions about their SRH. Rather than exercising individual agency, many women relied on healthcare providers, partners or family members to guide or fully make decisions regarding pregnancy, contraception and sexual relationships (Lundberg et al., 2012; Bagadia et al., 2020; Tumwakire et al., 2022; Rani et al., 2023; Vijayalakshmi et al., 2024a; Vijayalakshmi et al., 2024b).

This pattern of decreased autonomy extended to experiences where decisions were made with no regard to women’s knowledge or consent. In fact, some healthcare professionals reported making family planning decisions on behalf of their patients (Tumwakire et al., 2022). In other cases, women reported that both healthcare professionals and family members pushed sterilization as the most appropriate form of contraception (Vijayalakshmi et al., 2024b).

Women with SMI expressed emotional distress when their partners made decisions about their sexual health without consulting them (Rani et al., 2023). Economic dependence further exacerbated these power dynamics. When describing how her parents would not pay for her healthcare costs, a woman told researchers that she “paid” her clinician by non-consensually engaging in sexual activity with him (Lundberg et al., 2012).

Women also reported limited choice in the use of contraception (Bagadia et al., 2020; Vijayalakshmi et al., 2024a; Vijayalakshmi et al., 2024b). Although some women reported a general refusal to use contraception, saying that they are not worried about repercussions (Bagadia et al., 2020), others expressed that this decision is usually made by partners, detailing that their partner dislikes using any contraception (Vijayalakshmi et al., 2024b). One study found that family members sometimes stopped women from engaging in sexual intercourse entirely by limiting who they interacted with (Wainberg et al., 2007).

Despite knowing about both the emotional and physical violence perpetrated against women, many mental healthcare providers did not intervene in cases of inpatient violence, due to fear of patient aggression (Tumwakire et al., 2022) or concern that action against the abuser may result in consequences for the victim, such as abandonment or retribution (Rani et al., 2023).

##### Facilitators

A few studies found that women desire to share decision-making with their spouses. One participant in China expressed that she wished she could bear the risks of reproduction with the help of her husband (Yu et al., 2022). Women in Ethiopia with SMI also expressed positive attitudes toward, and agency over, contraceptive use (Zerihun et al., 2021).

#### Synthesized finding 4: Stigma and gendered expectations around mental illness and sexuality, reinforced by societal norms, discourage care-seeking for the SRH needs of women with SMI (Figure 5)

##### Barriers

Women with SMI often struggle to openly discuss their SRH needs due to cultural and familial expectations. Feelings of shame, discomfort and fear of disclosure emerged across studies as key deterrents to care-seeking (Raisi et al., 2018; Zerihun et al., 2021). One mental healthcare professional in Iran stated that patients only talk about SRH issues after a few sessions, and still caution, like a private setting, has to be used (Raisi et al., 2018). Many women in Iran also expressed not raising sexual inquiries due to cultural norms of abstinence before marriage, and Iranian mental healthcare professionals reiterated this social norm as a factor behind patients asking questions about SRH only after marriage (Raisi et al., 2018). Women also voiced fear about private SRH information being unduly disclosed to others by healthcare professionals (Raisi et al., 2018).Women also expressed concern that their baby will also have an SMI and therefore be labeled as “disabled” by society (Zerihun et al., 2021).

These cultural constraints were compounded by internalized norms around gender and sexuality. Many women reported that prescribed gender roles were more important than sexual desire, with one declaring that her children’s education was more important than her SRH needs (Vijayalakshmi et al., 2024b). Studies also reported women with SMI overlooking STI symptoms due to family issues, which they perceived to be more pressing (Vijayalakshmi et al., 2024a; Vijayalakshmi et al., 2024b).

Mental healthcare professionals tended to make assumptions about the capability of women with SMI; these attitudes often limited the reproductive choices and medical options women were given. Healthcare professionals often believed that women with SMI have decreased caretaking abilities, and such beliefs enabled them to administer family planning services to women with SMI without consent (Tumwakire et al., 2022). In one study, women with SMI reported disliking family planning, believing it was for preventing birth rather than planning it (Zerihun et al., 2021). Mental healthcare professionals also believed that the sexual habits of women with SMI were attributable to their SMI (Wainberg et al., 2007).

Lastly, the reproductive decision-making of women with SMI was heavily driven by expectations surrounding motherhood, often leading to delayed care or compromised autonomy. In one study, women with SMI reported letting familial opinions influence their decisions regarding SRH care, including contraception (Bagadia et al., 2020). Women with SMI in Ethiopia also expressed their experience that other people did not think that women with SMI should have children due to a perceived caretaking inability and the possibility of children inheriting SMI (Zerihun et al., 2021). Additionally, a familial expectation for male children in India was reported to lead to abortion of female fetuses and delayed sterilization of women (Bagadia et al., 2020).

##### Facilitators

Motherhood was reported as a powerful motivator for recovery in China, as it helped women with SMI feel similar to people without SMI. Women with SMI also exhibited positive attitudes toward SRH care, especially pre- and antenatal care, which they believe is technologically advanced and therefore reliable (Yu et al., 2022). Others reported feeling more comfortable pursuing sexual relationships within psychiatric treatment facilities (Wainberg et al., 2007).

Additionally, a mental healthcare professional reported women’s sex-positive attitudes when given a proper outlet, and a woman with SMI expressed a desire to learn more about STI prevention in order to educate her community (Wainberg et al., 2007).

### Confidence of findings

For dependability, all synthesized findings were informed by high-quality studies; however, because these studies contained limitations, dependability for all four synthesized findings was downgraded to “moderate.” For credibility, because most contributing findings were ranked as “unequivocal,” all synthesized findings were rated as “high.” As a result, all synthesized findings received a final “moderate” ConQual rating (see Supplementary File S5).

## Discussion

This review found that limited knowledge and service availability, women’s restricted autonomy and stigma and societal norms were intersecting factors that shape access to SRH care among women with SMI in LMICs. Our findings provide varying degrees of insight across the five dimensions of access.

*Approachability:* When considering approachability of services, a general lack of knowledge and reduced risk perception of SRH needs sometimes led to decreased perception of need for care. Women with SMI were sometimes not aware of STI symptoms or, even if they were aware, did not prioritize treatment due to a lack of concern for potential negative impacts or familial issues.

*Acceptability:* In terms of acceptability, social norms were both a barrier and facilitator to access. Some barriers, such as shame and stigma around discussing SRH needs, may reflect patterns in the general population (Cook and Dickens, 2014) – particularly among unmarried women (Mohammadi et al., 2016) or young people (Riabroi et al., 2025) – rather than phenomena unique to women with SMI. However, other barriers were more clearly tied to perceptions of SMI. For example, mental healthcare professionals and family members often perceived women with SMI to have reduced caretaking abilities. The lack of access to essential SRH care, particularly for women who are less educated, live in rural areas and are poorer, is a global phenomenon that reflects deep-rooted gender inequalities (Starrs et al., 2018). These gaps seem to be intensified further for women with SMI, echoing the experiences of other marginalized populations of women. For example, a global systematic review found that the femininity of women with intellectual disabilities was considered “defective” and that their caretakers considered them to be undeserving of perceived “adult” needs like sexual desires, limiting access to SRH care (Pérez-Curiel et al., 2023).

Interpersonal violence is mentioned in many of the papers in the form of gendered physical, sexual and emotional abuse that is perpetrated by partners, family or community members, healthcare workers and others in positions of power. However, despite the exacerbation of sexual violence among women with SMI (Lundberg et al., 2015), the studies in this review did not mention the impact that it may have on access to care. Experience of sexual violence is a health concern itself, and may have mental health, SRH and other physical health sequelae (Hillcoat et al., 2023). Previous research in the general population indicates that sexual assault may lead to avoidance or disengagement with gynecological care (Razi et al., 2021) and more rapid discontinuation of contraception (Allsworth et al., 2013). It is conceivable these experiences could have similar effects among women with SMI, but there was insufficient data in our review to form conclusions on this point. Although traditional and religious providers are commonly sought for mental health problems in LMIC (Asher et al., 2021), the included studies did not explore preferences for alternative sources of care for SRH among women with SMI.

*Availability*: Availability of SRH care varied across studies. Though it was not always explicitly stated, it seemed that in some cases these services were available, but were harder to access for women with SMI. A perceived gap was that mental healthcare professionals did not proactively ask about SRH, nor signpost to available services, and this was traced back to mental healthcare workers not feeling equipped – both in terms of training and time – to address SRH needs. There was less information on how practical issues such as geographic location, transport or opening hours affected access, and little exploration of how services could be tailored to the specific needs of women with SMI.

*Affordability*: The lack of emphasis on affordability in our findings may be because this was simply not the focus of the included papers. Out-of-pocket expenditure is a major barrier to accessing SRH services in LMICs among the general population (Ravindran and Govender, 2020); however, our study was not able to shed light on any differential impacts on women with SMI.

*Appropriateness*: There were several barriers relating to appropriateness of care. Mental healthcare providers attributed abnormal sexual behavior to SMI, not intervening in cases of sexual violence and in some cases seeming indifferent to the SRH needs of women. Additionally, women with SMI expressed fear that providers would disclose information to family members that would then change the way family members perceived them, making them more hesitant to access care. Engagement with care was highly shaped by lack of autonomy in decision-making. People with SMI in LMICs may be uninvolved in decision-making in general (Souraya et al., 2018), but also women in LMICs have low decision-making autonomy in relation to SRH, such as contraceptive use (Belachew et al., 2023). Women with SMI in LMICs, ergo, face intersectional layers of disempowerment – stemming from gendered discrimination, SMI stigma and lack of resources in health systems – that lead to decreased autonomy over SRH care decisions.

### Recommendations

We propose the following policy and practice recommendations on the basis of our findings.

#### Synthesized finding 1: Variable knowledge of SRH and low risk perception hinder access to SRH care for women with SMI

The majority of evaluations aiming to improve SRH in women with SMI are health promotion interventions focused on changing women’s awareness, skills and behaviors in relation to SRH. To our knowledge, all evaluations have been conducted in the US or UK (Pandor et al., 2015; Hughes et al., 2020) and the overall quality of evidence is limited. Although similar interventions could be adapted or developed to suit LMIC settings, our findings suggest focusing on service user factors may be necessary but not sufficient to improve access to SRH care.

#### Synthesized finding 2: Service availability shapes access to SRH care for women with SMI

The inclusion of essential SRH services in universal health coverage is a crucial step to increasing access for all women (Starrs et al., 2018). Further, addressing the specific provider-side factors affecting access for women with SMI may involve training mental healthcare professionals, and other health workers involved in delivering mental healthcare (for example, community health workers) in the SRH needs of women with SMI. Training could be modeled on that developed for healthcare professionals working with similarly vulnerable populations, like refugee women (Mengesha et al., 2018). Local guidelines to ensure routine assessment and referral for SRH needs are also indicated. Using mHealth innovations to deliver SRH care could conceivably address limitations of healthcare worker’s workload by spreading care demand, and increased anonymity could combat the shame both patients and mental healthcare professionals reported by allowing non-face-to-face treatment (Laar et al., 2022). However, this may not serve the entire population due to gaps in literacy or digital access (Alkureishi et al., 2021), and our data did not speak to the acceptability or feasibility of an mHealth approach.

Global guidance may also be important: while the WHO Guidance on Community Mental Health Services (WHO, 2021) and WHO QualityRights Initiative (WHO, 2019b) seek to support mental health services to align with the CRPD, there is limited guidance on how SRH needs can be addressed among people with SMI. The WHO’s Mental Health Gap Intervention Guide also does not signpost clinicians to routinely consider SRH concerns, other than sexual abuse, dysfunction and infection, in women with SMI (WHO, 2019a).

#### Synthesized finding 3: Restricted autonomy and exclusion from health decisions prevent women with SMI from accessing SRH care that reflects their needs and rights

Studies in some contexts have shown that autonomy is positively associated with healthcare decision-making and better health outcomes, especially in SRH (Osamor and Grady, 2016). However, in other contexts, family decision-making is expected and disregarding these norms could result in worse outcomes. For example, unilateral decision-making on contraception could conceivably rupture intimate relationships or even lead to harm or abuse. Care models that actively support and foster women’s agency should therefore be developed while paying attention to cultural and social norms. One approach is to employ recovery-oriented models of care that center on personal goals and restoration of autonomy, while being grounded in positive family relationships (Asher et al., 2022; Asher et al., 2024; Brooke-Sumner et al., 2024). Because autonomy in LMICs is often dictated by communality rather than individuality (Mumtaz and Salway, 2009), community health workers who have intimate knowledge about social spheres and the marginalized populations within them (Ahmed et al., 2022) can act as key facilitators in supporting contextually appropriate expressions of autonomy. However, confidentiality concerns arising from the close social and geographical proximity of local community health workers providing SRH services (Sidamo et al., 2024) might be amplified in settings where the prevailing belief is that women with SMI should not want or need access to SRH care. Such contextual sensitivities should be actively identified and addressed when developing interventions.

#### Synthesized finding 4: Stigma and gendered expectations around mental illness and sexuality, reinforced by societal norms, discourage care-seeking for the SRH needs of women with SMI

The normalization of both SMI and SRH care is essential in combating societal stigma. Social contact interventions have been shown to decrease mental health stigma in LMICs (Makhmud et al., 2022). Healthcare workers hearing directly from women with SMI about their experience of SRH may have an important contribution to changing attitudes and willingness to address SRH concerns. Family members had a critical role in shaping the sexual relationships and access to SRH care of women SMI. Work with families may therefore be important in changing expectations and attitudes in relation to SRH, while carefully navigating contextual gender norms. This might include partners, parents or other family members, who are typically heavily involved in care of people with SMI in many LMIC (Dijkstra et al., 2025; Rapiya et al., 2025). Broader policy and legislative changes that promote gender equality and give women more control over their lives are also needed to shift social norms that impede women’s access to SRH care (Starrs et al., 2018). These reforms are likely to sit outside the health sector and include education, economic policy, community and civil society, and the criminal justice system (Sarri et al., 2025).

### Research recommendations

More research is needed to understand the interactions between enduring emotional, physical and sexual abuse and its impact on accessing SRH care. Research must also be done on the affordability and feasibility of accessing SRH care, including transport and location of care among women with SMI. Interrogation of the extent to which problems with access to SRH among women with SMI reflect access gaps in the general population could also be a focus. Lastly, development and evaluation of interventions to address SRH – focusing on women, families, health systems and broader social, cultural and economic structures – is needed.

### Strengths and limitations

Rigorously applying the JBI meta-aggregation approach facilitates the formation of recommendations that can be readily translated into public health interventions. Further, using ConQual increases confidence in the validity of our synthesized findings. However, pooling findings by meta-aggregation may overlook important contextual differences between countries, including cultural and social norms and health service availability. Furthermore, as only six countries were represented, we should be wary of generalizing our findings to diverse LMIC contexts. Additionally, as most studies were set in specialized psychiatric settings, our findings may not be generalizable to community or primary care settings. The majority of screening was carried out by a single reviewer, which may increase the possibility of incorrect assessment and missing data.

## Conclusion

This review synthesized the barriers and facilitators to SRH with women with SMI in LMICs, identifying four main addressable findings that serve as influencers to access. These findings can help policymakers in making targeted interventions to improve access to SRH in these settings. Differences in cultural and social context and healthcare systems must be considered before adopting interventions.

## Supporting information

10.1017/gmh.2026.10222.sm001 Chalmeti et al. supplementary material Chalmeti et al. supplementary material

## Data Availability

Data sharing is not applicable – no new data is generated.

## Supplementary material

The supplementary material for this article can be found at http://doi.org/10.1017/gmh.2026.10222.

## Inclusivity statement

In this article, the term “women” is used to refer to study participants who were individuals who self-identified as women within the socially defined gender norms of their country.

## Figure 1. Long description

At the top, two boxes: left, Studies from databases/registers n equals 6,906, broken down as Embase n equals 3,260, Psyc I N F O n equals 2,096, M E D L I N E n equals 1,550; right, References from other sources n equals 7, Citation searching n equals 7. Both feed into References removed n equals 2,219, with Duplicates identified manually n equals 342, Duplicates identified by Covidence n equals 1,877, Marked as ineligible by automation tools n equals 0, Other reasons n equals 0. The remaining Studies screened n equals 4,694, with a right branch for Studies excluded n equals 4,594. Downward, Studies sought for retrieval n equals 100, with a right branch for Studies not retrieved n equals 0. Next, Studies assessed for eligibility n equals 100, with a right branch for Studies excluded n equals 89, detailed as Ineligible setting n equals 3, Different outcomes n equals 32, Comparator study n equals 1, Ineligible indication n equals 9, Conference paper n equals 4, Publication year n equals 3, Ineligible intervention n equals 8, Ineligible study design n equals 26, Developed countries n equals 3. The final box at the bottom is Studies included in review n equals 11. A dashed box below notes Included studies ongoing n equals 0, Studies awaiting classification n equals 0.

Navigate back to Figure 1..

## Table 1. Long description

Column headers from left to right are Paper, Country and Setting, Study design, Phenomena of interest, Participant characteristics, Sampling methods, Data collection and analytical method, and Reported limitations. The first row details Bagadia et al., 2020, India, perinatal psychiatric service, qualitative study, factors influencing pregnancy decision-making in women with S M I, women aged 22 to 36 years, n equals 42, purposive sampling, semi-structured interviews with grounded theory, limitation is sample from perinatal psychiatric service not generalizable. The second row is Lundberg et al., 2012, Uganda, psychiatric inpatient and outpatient services, qualitative study, S M I influence on sexual risk behaviors and abuse, people aged 18 to 49 years, n equals 20, purposive sampling, semi-structured interviews with content analysis, limitation is participant passivity and reduced speech. The third row is Raisi et al., 2018, Iran, psychiatric hospital, qualitative study, S R H needs of Iranian patients with mental illness, people with S M I, family and healthcare professionals aged 18 years and older, n equals 17, purposive sampling, in-depth semi-structured interviews with content analysis, limitation is incomplete disclosure of issues. The fourth row is Rani et al., 2023, India, tertiary care mental health institution, qualitative study, views on abuse experienced by people with S M I, people with S M I, experts, caregivers aged 18 years and older, n equals 51, purposive sampling, focus group discussions with thematic analysis, limitation is lack of generalizability to ethnically diverse populations. The fifth row is Rezaie and Phillips, 2020, Iran, psychiatric inpatient unit, qualitative study, post-discharge needs of women with S M I, women aged 40 years and older, n equals 42, purposive sampling, focus group interviews with conventional content analysis, limitation is only patient perspective included. The sixth row is Tumwakire et al., 2022, Uganda, mental referral hospital, qualitative study, mental healthcare workers’ perspectives on S R H needs, mental health workers aged 18 years and older, n equals 14, purposive sampling, semi-structured and in-depth interviews with thematic content analysis and inductive approach, limitation is single site. The seventh row is Vijayalakshmi et al., 2024a, India, psychiatric outpatient clinic, qualitative study, S R H needs of women with S M I, women of reproductive age with S M I aged 18 to 49 years, n equals 32, purposive sampling, in-depth face-to-face interview with Hycner's explication, limitation is focus on women with sexual dysfunction and S T I symptoms in single setting. The eighth row is Vijayalakshmi et al., 2024b, India, tertiary care psychiatric hospitals, integrated mixed-method study, S R H needs of women with S M I, women aged 18 to 49 years, n equals 32, purposive sampling, semi-structured interviews with Hycner's explication process, limitation is limited generalizability and unexplored sexual dysfunction and psychiatric medications. The ninth row is Wainberg et al., 2007, Brazil, psychiatric institutions, ethnography study, application of interventions developed in the United States, patients aged 18 to 65 years, n equals 45, purposive sampling, field observations, focus groups, key-informant interviews, grounded theory, limitation is limited relevance of U S interventions. The tenth row is Yu et al., 2022, China, psychiatric hospital, descriptive phenomenology study, S R H needs of women with schizophrenia, women aged 26 to 40 years, n equals 15, purposive sampling, semi-structured and in-depth interviews with Colaizzi's descriptive analysis, limitation is exclusion of women without desire for children. The eleventh row is Zerihun et al., 2021, Ethiopia, women with S M I in population-based cohort study, qualitative study, unmet family planning needs, women aged 18 to 49 years, n equals 16, purposive sampling, in-depth interviews with thematic analysis and inductive approach, limitation is participants had better access to mental services than general population.

Navigate back to Table 1..

## Figure 2. Long description

From left to right, the leftmost column labeled Barriers contains seven red rounded rectangles stacked vertically: Beliefs about relationship between mental healthcare and S R H, Beliefs about impact of medication on S R H, Risk-taking behaviors despite knowledge of risks, No perceived need for sex education by women with S M I, Lack of knowledge of S T I symptoms and treatment amongst women with S M I, Poor knowledge of contraception amongst women with S M I, and Condoms viewed negatively. The central gray circle states Variable knowledge of S R H and low risk perception hinder access to S R H care for women with S M I. The rightmost column labeled Facilitators contains two green rounded rectangles stacked vertically: General awareness regarding H I V amongst women with S M I, and Inclusive and supportive learning environment for women's S R H education.

Navigate back to Figure 2..

## Figure 3. Long description

At the center is a grey circle labeled Service availability shapes access to S R H care for women with S M I. To the left, under Barriers, four red boxes list Lack of time for mental healthcare professionals to address S R H, Lack of policies on how to address S R H in mental healthcare facilities, Lack of family planning services, and Lack of S R H skills and knowledge amongst mental healthcare professionals. To the right, under Facilitators, two green boxes list Access to S R H services is wanted by women with S M I and Mental healthcare professionals perceive women with S M I to have similar S R H needs to people without S M I.

Navigate back to Figure 3..

## Figure 4. Long description

On the left, under the heading Barriers, nine red boxes are stacked vertically: stigma around mental illness, gender preference for baby, mental healthcare professionals feel uncomfortable discussing S R H, women with S M I feel shame when talking about S R H, concern about disclosure, women with S M I are perceived to be incapable of raising children, gendered family roles prioritized over S R H concerns, mental healthcare professionals assume sexual behaviors are due to S M I, misunderstanding around family planning services. At the center, a gray circle states that stigma and gendered expectations around mental illness and sexuality, reinforced by societal norms, discourage care-seeking for the S R H needs of women with S M I. On the right, under the heading Facilitators, three green boxes are stacked vertically: opportunities for peer education on S R H valued by women with S M I, sex-positive attitudes and environment, perceived rewards of motherhood.

Navigate back to Figure 4..
