# Supplementary material for: Barriers and facilitators to sexual and reproductive healthcare access for women with severe mental illness in low- and middle-income countries: A qualitative systematic review and meta-aggregation
Source: Glob Ment Health (Camb). 2026 May 20;13:e119. doi: 10.1017/gmh.2026.10222 (PMC13279974; doi:10.1017/gmh.2026.10222)
Supplement: Chalmeti et al. supplementary material [file S2054425126102222sup001.zip › Supplementary File 4.docx]

**ConQual Ratings**

| **Synthesized Finding** | **Contributing Studies** | **Dependability** | **Credibility** | **ConQual Rating** |
| --- | --- | --- | --- | --- |
| *SF1:* Limited knowledge of SRH, low risk perception, and poor service availability hinder access to SRH care for women with SMI. | (Bagadia et al., 2020, Yu et al., 2022, Rezaie and Phillips, 2020, Vijayalakshmi et al., 2024b, Vijayalakshmi et al., 2024a, Raisi et al., 2018, Tumwakire et al., 2022, Wainberg et al., 2007, Zerihun et al., 2021) | Moderate | High | Moderate |
| *SF2:* Restricted autonomy and exclusion from health decisions prevent women with SMI from accessing SRH care that reflects their needs and rights. | (Bagadia et al., 2020, Rani et al., 2023, Tumwakire et al., 2022, Vijayalakshmi et al., 2024a, Vijayalakshmi et al., 2024b, Wainberg et al., 2007, Yu et al., 2022, Zerihun et al., 2021, Lundberg et al., 2012) | Moderate | High | Moderate |
| *SF3:* Stigma around mental illness and sexuality, reinforced by societal norms, discourages care-seeking for the SRH needs of women with SMI. | (Bagadia et al., 2020, Raisi et al., 2018, Tumwakire et al., 2022, Vijayalakshmi et al., 2024a, Vijayalakshmi et al., 2024b, Wainberg et al., 2007, Yu et al., 2022, Zerihun et al., 2021) | Moderate | High | Moderate |
