# Supplementary material for: Barriers and facilitators to sexual and reproductive healthcare access for women with severe mental illness in low- and middle-income countries: A qualitative systematic review and meta-aggregation
Source: Glob Ment Health (Camb). 2026 May 20;13:e119. doi: 10.1017/gmh.2026.10222 (PMC13279974; doi:10.1017/gmh.2026.10222)
Supplement: Chalmeti et al. supplementary material [file S2054425126102222sup001.zip › Supplementary File 2.docx]

**Ovid MEDLINE**

2004 to July 2024; 1550 results

| 1. | ("mental disorder*" or "mental illness" or "severe mental illness" or "psychiatric illness" or "psychiatric disease" or SMI or schizophrenia or bipolar or psychosis or "Depression with Psychotic symptom*" or "psychotic disorder*").mp. [mp=title, book title, abstract, original title, name of substance word, subject heading word, floating sub-heading word, keyword heading word, organism supplementary concept word, protocol supplementary concept word, rare disease supplementary concept word, unique identifier, synonyms, population supplementary concept word, anatomy supplementary concept word] |
| --- | --- |
| 2. | ("sexual health" or "reproductive health" or "sexual healthcare" or SRH or "reproductive healthcare*" or "reproductive health service*" or "family planning" or "gynecological issue*" or "sexual health service*" or "HIV Infection*" or "HIV Screening" or "STD screening" or "sexually transmitted infection*" or sexuality or 'women's health' or conception or contraception or fertility or postpartum or "Maternal Health" or "sexual violence" or "sexual abuse" or "sexual trauma" or "sexual assault" or "equality to access SRH services" or "sexual and reproductive health").mp. [mp=title, book title, abstract, original title, name of substance word, subject heading word, floating sub-heading word, keyword heading word, organism supplementary concept word, protocol supplementary concept word, rare disease supplementary concept word, unique identifier, synonyms, population supplementary concept word, anatomy supplementary concept word] |
| 3. | (afghan* or africa* or albania* or algeria* or angola* or antigua* or barbuda* or argentin* or armenia* or aruba* or azerbaijan* or bahrain* or bangladesh* or bengal* or bangal* or barbad* or bajan* or belarus* or belorus* or byelarus* or byelorus* or belize* or benin* or dahomey or bhutan* or bolivia* or bosnia* or herzegovin* or botswan* or batswan* or bechuanaland* or brazil* or brasil* or bulgaria* or burkin* or upper volta* or burundi* or urundi* or "cabo verde*" or cape verde* or cambodia* or kampuchea* or khmer* or cameroon* or cameroun* or "ubangi shari*" or chad* or chile* or chin* or colombia* or comor* or mayotte* or congo* or zaire* or "costa rica*" or "cote d'ivoir*" or "cote d' ivoir*" or "cote divoir*" or "cote d ivoir*" or ivory coast* or ivorian* or croatia* or cuba* or cyprus* or cypriot* or czech* or djibouti* or "french somaliland*" or dominica* or ecuador* or egypt* or united arab republic* or el salvador* or salvadoran* or guinea* or equatoguinea* or eritrea* or estonia* or eswatini* or swaziland* or swazi* or swati* or ethiopia* or fiji* or gabon* or gabonese* or gabonaise* or gambia* or ((georgia or georgian or georgians) not (atlanta or california or florida)) or ghana* or gibraltar* or greece* or greek* or grecian* or grenada* or grenadian* or guam* or guatemala* or guyan* or guiana* or haiti* or hispaniola* or hondura* or hungar* or india* or indonesia* or iran* or iraq* or "isle of man" or jamaica* or jordan* or kazakh* or kenya* or karabati* or korea* or kosovo* or kosova* or kyrgyz* or kirgiz* or kirghiz* or laos or lao or laotian* or latvia* or lebanon* or lebanese* or lesotho* or lesothan* or lesothonia* or basutoland* or mosotho* or basotho* or liberia* or libya* or jamahiriya* or lithuania* or macedonia* or madagasca* or malagasy* or malawi* or nyasaland* or malaysia* or "malay* federation" or maldives* or maldivian* or indian ocean or mali or malian* or malta or maltese* or micronesia* or marshallese* or kiribati* or marshall island* or nauru or nauran or nauruans or mariana or marianas or palau or paluan* or tuvalu* or mauritania* or mauritan* or mauritius* or mexico* or mexican* or moldova* or moldovia* or mongol* or montenegr* or morocc* or ifni or mozambique or mozambican* or myanmar* or burma* or burmese or namibia* or nepal* or "new caledonia*" or "netherlands antill*" or nicaragua* or niger* or oman* or pakistan* or palestin* or gaza* or west bank* or panama* or paraguay* or peru or peruvian* or "peru's" or philippine* or philipine* or phillipine* or phillippine* or filipino* or filipina* or poland* or polish or pole* or portugal* or portuguese or puerto ric* or romania* or russia* or ussr* or soviet* or rwanda* or rwandese or ruanda* or ruandese or samoa* or navigator island* or pacific island* or polynesia* or "sao tome and principe*" or "sao tomean*" or santomean* or "saudi arabia*" or saudi or saudis or senegal* or serbia* or seychell* or "sierra leone*" or slovak* or sloven* or melanesia* or solomon island* or norfolk island* or somali* or "sri lanka*" or ceylon* or "saint kitts and nevis*" or "st kitts and nevis*" or kittian* or nevisian* or "saint lucia*" or "st lucia*" or "saint vincent*" or "st vincent*" or vincentian* or grenadine* or sudan* or surinam* or syria* or tajik* or tadjik* or tadzhik* or tanzania* or tanganyika* or thai* or "timor leste*" or "east timor*" or timorese* or togo or togoles or tonga* or trinidad* or tobago* or tunisia* or turkiy* or turkey* or turk* or turkmen* or uganda* or ukrain* or uruguay* or uzbek* or vanuatu* or "new hebrides*" or venezuela* or vietnam* or "viet nam*" or yemen* or yugoslav* or zambia* or zimbabwe* or rhodesia* or "arab* countr*" or "middle east*" or "global south" or sahara* or subsahara* or magreb* or maghrib* or "west indie*" or caribbean* or "central america*" or "latin america*" or "south america*" or "central asia*" or "north asia*" or "northern asia*" or "southeastern asia*" or "south eastern asia*" or "southeast asia*" or "south east asia*" or "west asia*" or "western asia*" or "east europe*" or "eastern europe*" or "developing countr*" or "developing nation*" or "developing population*" or "developing world" or "less developed countr*" or "less developed nation*" or "less developed world" or "lesser developed countr*" or "lesser developed nation*" or "lesser developed world" or "under developed countr*" or "under developed nation*" or "under developed world" or "underdeveloped countr*" or "underdeveloped nation*" or "underdeveloped world" or "middle income countr*" or "middle income nation*" or "middle income population*" or "low income countr*" or "low income nation*" or "low income population*" or "lower income countr*" or "lower income nation*" or "lower income population" or "underserved countr*" or "underserved nation*" or "underserved population" or "under served population" or "under served nation*" or "under served population" or "deprived countr*" or "deprived population" or "high burden countr*" or "high burden nation*" or "countdown countr*" or "countdown nation*" or "poor countr*" or "poor nation*" or "poor population" or "poor world" or "poorer countr*" or "poorer nation*" or "poorer population" or "poorer world" or "developing econom*" or "less developed econom*" or "underdeveloped econom*" or "under developed econom*" or "middle income econom*" or "low income econom*" or "lower income econom*" or "low gdp" or "low gnp" or "low gross domestic" or "low gross national" or "lower gdp" or "lower gnp" or "lower gross domestic" or "lower gross national" or lmic or lmics or "third world" or "lami countr*" or "transitional countr*" or "emerging econom*" or "emerging nation*").mp. [mp=title, book title, abstract, original title, name of substance word, subject heading word, floating sub-heading word, keyword heading word, organism supplementary concept word, protocol supplementary concept word, rare disease supplementary concept word, unique identifier, synonyms, population supplementary concept word, anatomy supplementary concept word] |
| 4. | 1 and 2 and 3 |
| 5. | limit 4 to (english language and yr="2004 - 2024" and "all adult (19 plus years)" and english and last 20 years) |
| 6. | limit 5 to yr="2004 - 2024" |

**Ovid EMBASE**

2004 to July 2024; 3260 results

| 1. | ("mental disorder*" or "mental illness" or "severe mental illness" or "psychiatric illness" or "psychiatric disease" or SMI or schizophrenia or bipolar or psychosis or "Depression with Psychotic symptom*" or "psychotic disorder*").mp. [mp=title, abstract, heading word, drug trade name, original title, device manufacturer, drug manufacturer, device trade name, keyword heading word, floating subheading word, candidate term word] |
| --- | --- |
| 2. | ("sexual health" or "reproductive health" or "sexual healthcare" or SRH or "reproductive healthcare*" or "reproductive health service*" or "family planning" or "gynecological issue*" or "sexual health service*" or "HIV Infection*" or "HIV Screening" or "STD screening" or "sexually transmitted infection*" or sexuality or 'women's health' or conception or contraception or fertility or postpartum or "Maternal Health" or "sexual violence" or "sexual abuse" or "sexual trauma" or "sexual assault" or "equality to access SRH services" or "sexual and reproductive health").mp. [mp=title, abstract, heading word, drug trade name, original title, device manufacturer, drug manufacturer, device trade name, keyword heading word, floating subheading word, candidate term word] |
| 3. | (afghan* or africa* or albania* or algeria* or angola* or antigua* or barbuda* or argentin* or armenia* or aruba* or azerbaijan* or bahrain* or bangladesh* or bengal* or bangal* or barbad* or bajan* or belarus* or belorus* or byelarus* or byelorus* or belize* or benin* or dahomey or bhutan* or bolivia* or bosnia* or herzegovin* or botswan* or batswan* or bechuanaland* or brazil* or brasil* or bulgaria* or burkin* or upper volta* or burundi* or urundi* or "cabo verde*" or cape verde* or cambodia* or kampuchea* or khmer* or cameroon* or cameroun* or "ubangi shari*" or chad* or chile* or chin* or colombia* or comor* or mayotte* or congo* or zaire* or "costa rica*" or "cote d'ivoir*" or "cote d' ivoir*" or "cote divoir*" or "cote d ivoir*" or ivory coast* or ivorian* or croatia* or cuba* or cyprus* or cypriot* or czech* or djibouti* or "french somaliland*" or dominica* or ecuador* or egypt* or united arab republic* or el salvador* or salvadoran* or guinea* or equatoguinea* or eritrea* or estonia* or eswatini* or swaziland* or swazi* or swati* or ethiopia* or fiji* or gabon* or gabonese* or gabonaise* or gambia* or ((georgia or georgian or georgians) not (atlanta or california or florida)) or ghana* or gibraltar* or greece* or greek* or grecian* or grenada* or grenadian* or guam* or guatemala* or guyan* or guiana* or haiti* or hispaniola* or hondura* or hungar* or india* or indonesia* or iran* or iraq* or "isle of man" or jamaica* or jordan* or kazakh* or kenya* or karabati* or korea* or kosovo* or kosova* or kyrgyz* or kirgiz* or kirghiz* or laos or lao or laotian* or latvia* or lebanon* or lebanese* or lesotho* or lesothan* or lesothonia* or basutoland* or mosotho* or basotho* or liberia* or libya* or jamahiriya* or lithuania* or macedonia* or madagasca* or malagasy* or malawi* or nyasaland* or malaysia* or "malay* federation" or maldives* or maldivian* or indian ocean or mali or malian* or malta or maltese* or micronesia* or marshallese* or kiribati* or marshall island* or nauru or nauran or nauruans or mariana or marianas or palau or paluan* or tuvalu* or mauritania* or mauritan* or mauritius* or mexico* or mexican* or moldova* or moldovia* or mongol* or montenegr* or morocc* or ifni or mozambique or mozambican* or myanmar* or burma* or burmese or namibia* or nepal* or "new caledonia*" or "netherlands antill*" or nicaragua* or niger* or oman* or pakistan* or palestin* or gaza* or west bank* or panama* or paraguay* or peru or peruvian* or "peru's" or philippine* or philipine* or phillipine* or phillippine* or filipino* or filipina* or poland* or polish or pole* or portugal* or portuguese or puerto ric* or romania* or russia* or ussr* or soviet* or rwanda* or rwandese or ruanda* or ruandese or samoa* or navigator island* or pacific island* or polynesia* or "sao tome and principe*" or "sao tomean*" or santomean* or "saudi arabia*" or saudi or saudis or senegal* or serbia* or seychell* or "sierra leone*" or slovak* or sloven* or melanesia* or solomon island* or norfolk island* or somali* or "sri lanka*" or ceylon* or "saint kitts and nevis*" or "st kitts and nevis*" or kittian* or nevisian* or "saint lucia*" or "st lucia*" or "saint vincent*" or "st vincent*" or vincentian* or grenadine* or sudan* or surinam* or syria* or tajik* or tadjik* or tadzhik* or tanzania* or tanganyika* or thai* or "timor leste*" or "east timor*" or timorese* or togo or togoles or tonga* or trinidad* or tobago* or tunisia* or turkiy* or turkey* or turk* or turkmen* or uganda* or ukrain* or uruguay* or uzbek* or vanuatu* or "new hebrides*" or venezuela* or vietnam* or "viet nam*" or yemen* or yugoslav* or zambia* or zimbabwe* or rhodesia* or "arab* countr*" or "middle east*" or "global south" or sahara* or subsahara* or magreb* or maghrib* or "west indie*" or caribbean* or "central america*" or "latin america*" or "south america*" or "central asia*" or "north asia*" or "northern asia*" or "southeastern asia*" or "south eastern asia*" or "southeast asia*" or "south east asia*" or "west asia*" or "western asia*" or "east europe*" or "eastern europe*" or "developing countr*" or "developing nation*" or "developing population*" or "developing world" or "less developed countr*" or "less developed nation*" or "less developed world" or "lesser developed countr*" or "lesser developed nation*" or "lesser developed world" or "under developed countr*" or "under developed nation*" or "under developed world" or "underdeveloped countr*" or "underdeveloped nation*" or "underdeveloped world" or "middle income countr*" or "middle income nation*" or "middle income population*" or "low income countr*" or "low income nation*" or "low income population*" or "lower income countr*" or "lower income nation*" or "lower income population" or "underserved countr*" or "underserved nation*" or "underserved population" or "under served population" or "under served nation*" or "under served population" or "deprived countr*" or "deprived population" or "high burden countr*" or "high burden nation*" or "countdown countr*" or "countdown nation*" or "poor countr*" or "poor nation*" or "poor population" or "poor world" or "poorer countr*" or "poorer nation*" or "poorer population" or "poorer world" or "developing econom*" or "less developed econom*" or "underdeveloped econom*" or "under developed econom*" or "middle income econom*" or "low income econom*" or "lower income econom*" or "low gdp" or "low gnp" or "low gross domestic" or "low gross national" or "lower gdp" or "lower gnp" or "lower gross domestic" or "lower gross national" or lmic or lmics or "third world" or "lami countr*" or "transitional countr*" or "emerging econom*" or "emerging nation*").mp. [mp=title, abstract, heading word, drug trade name, original title, device manufacturer, drug manufacturer, device trade name, keyword heading word, floating subheading word, candidate term word] |
| 4. | 1 and 2 and 3 |
| 5. | limit 4 to (english language and yr="2004 - 2024" and "all adult (19 plus years)" and english and last 20 years) |

**Ovid PsycINFO**

2004 to July 2024; 2096 results

| 1. | (Barrier* or restrict* or challenge* or obstacle*).mp. [mp=title, abstract, heading word, table of contents, key concepts, original title, tests & measures, mesh word] |
| --- | --- |
| 2. | (facilitat* or encourag*).mp. [mp=title, abstract, heading word, table of contents, key concepts, original title, tests & measures, mesh word] |
| 3. | 1 and 2 |
| 4. | (woman or women or female* or male* or men or girl* or lad* or "both gender*").mp. [mp=title, abstract, heading word, table of contents, key concepts, original title, tests & measures, mesh word] |
| 5. | mental disorders/ or "schizophrenia spectrum and other psychotic disorders"/ |
| 6. | ("mental disorder*" or "mental illness" or "severe mental illness" or SMI or schizophrenia or bipolar or psychosis or "Depression with Psychotic symptom*" or "psychotic disorder*").mp. [mp=title, abstract, heading word, table of contents, key concepts, original title, tests & measures, mesh word] |
| 7. | (access* or approach*).mp. [mp=title, abstract, heading word, table of contents, key concepts, original title, tests & measures, mesh word] |
| 8. | ("sexual health" or "reproductive health" or "sexual healthcare" or SRH or "reproductive healthcare*" or "reproductive health service*" or "family planning" or "gynecological issue*" or "sexual health service*").mp. [mp=title, abstract, heading word, table of contents, key concepts, original title, tests & measures, mesh word] |
| 9. | 4 and 5 and 6 |
| 10. | 5 or 6 |
| 11. | 4 and 10 |
| 12. | 3 and 11 |
| 13. | 8 and 12 |
| 14. | (afghan* or africa* or albania* or algeria* or angola* or antigua* or barbuda* or argentin* or armenia* or aruba* or azerbaijan* or bahrain* or bangladesh* or bengal* or bangal* or barbad* or bajan* or belarus* or belorus* or byelarus* or byelorus* or belize* or benin* or dahomey or bhutan* or bolivia* or bosnia* or herzegovin* or botswan* or batswan* or bechuanaland* or brazil* or brasil* or bulgaria* or burkin* or upper volta* or burundi* or urundi* or "cabo verde*" or cape verde* or cambodia* or kampuchea* or khmer* or cameroon* or cameroun* or "ubangi shari*" or chad* or chile* or chin* or colombia* or comor* or mayotte* or congo* or zaire* or "costa rica*" or "cote d'ivoir*" or "cote d' ivoir*" or "cote divoir*" or "cote d ivoir*" or ivory coast* or ivorian* or croatia* or cuba* or cyprus* or cypriot* or czech* or djibouti* or "french somaliland*" or dominica* or ecuador* or egypt* or united arab republic* or el salvador* or salvadoran* or guinea* or equatoguinea* or eritrea* or estonia* or eswatini* or swaziland* or swazi* or swati* or ethiopia* or fiji* or gabon* or gabonese* or gabonaise* or gambia* or ((georgia or georgian or georgians) not (atlanta or california or florida)) or ghana* or gibraltar* or greece* or greek* or grecian* or grenada* or grenadian* or guam* or guatemala* or guyan* or guiana* or haiti* or hispaniola* or hondura* or hungar* or india* or indonesia* or iran* or iraq* or "isle of man" or jamaica* or jordan* or kazakh* or kenya* or karabati* or korea* or kosovo* or kosova* or kyrgyz* or kirgiz* or kirghiz* or laos or lao or laotian* or latvia* or lebanon* or lebanese* or lesotho* or lesothan* or lesothonia* or basutoland* or mosotho* or basotho* or liberia* or libya* or jamahiriya* or lithuania* or macedonia* or madagasca* or malagasy* or malawi* or nyasaland* or malaysia* or "malay* federation" or maldives* or maldivian* or indian ocean or mali or malian* or malta or maltese* or micronesia* or marshallese* or kiribati* or marshall island* or nauru or nauran or nauruans or mariana or marianas or palau or paluan* or tuvalu* or mauritania* or mauritan* or mauritius* or mexico* or mexican* or moldova* or moldovia* or mongol* or montenegr* or morocc* or ifni or mozambique or mozambican* or myanmar* or burma* or burmese or namibia* or nepal* or "new caledonia*" or "netherlands antill*" or nicaragua* or niger* or oman* or pakistan* or palestin* or gaza* or west bank* or panama* or paraguay* or peru or peruvian* or "peru's" or philippine* or philipine* or phillipine* or phillippine* or filipino* or filipina* or poland* or polish or pole* or portugal* or portuguese or puerto ric* or romania* or russia* or ussr* or soviet* or rwanda* or rwandese or ruanda* or ruandese or samoa* or navigator island* or pacific island* or polynesia* or "sao tome and principe*" or "sao tomean*" or santomean* or "saudi arabia*" or saudi or saudis or senegal* or serbia* or seychell* or "sierra leone*" or slovak* or sloven* or melanesia* or solomon island* or norfolk island* or somali* or "sri lanka*" or ceylon* or "saint kitts and nevis*" or "st kitts and nevis*" or kittian* or nevisian* or "saint lucia*" or "st lucia*" or "saint vincent*" or "st vincent*" or vincentian* or grenadine* or sudan* or surinam* or syria* or tajik* or tadjik* or tadzhik* or tanzania* or tanganyika* or thai* or "timor leste*" or "east timor*" or timorese* or togo or togoles or tonga* or trinidad* or tobago* or tunisia* or turkiy* or turkey* or turk* or turkmen* or uganda* or ukrain* or uruguay* or uzbek* or vanuatu* or "new hebrides*" or venezuela* or vietnam* or "viet nam*" or yemen* or yugoslav* or zambia* or zimbabwe* or rhodesia* or "arab* countr*" or "middle east*" or "global south" or sahara* or subsahara* or magreb* or maghrib* or "west indie*" or caribbean* or "central america*" or "latin america*" or "south america*" or "central asia*" or "north asia*" or "northern asia*" or "southeastern asia*" or "south eastern asia*" or "southeast asia*" or "south east asia*" or "west asia*" or "western asia*" or "east europe*" or "eastern europe*" or "developing countr*" or "developing nation*" or "developing population*" or "developing world" or "less developed countr*" or "less developed nation*" or "less developed world" or "lesser developed countr*" or "lesser developed nation*" or "lesser developed world" or "under developed countr*" or "under developed nation*" or "under developed world" or "underdeveloped countr*" or "underdeveloped nation*" or "underdeveloped world" or "middle income countr*" or "middle income nation*" or "middle income population*" or "low income countr*" or "low income nation*" or "low income population*" or "lower income countr*" or "lower income nation*" or "lower income population" or "underserved countr*" or "underserved nation*" or "underserved population" or "under served population" or "under served nation*" or "under served population" or "deprived countr*" or "deprived population" or "high burden countr*" or "high burden nation*" or "countdown countr*" or "countdown nation*" or "poor countr*" or "poor nation*" or "poor population" or "poor world" or "poorer countr*" or "poorer nation*" or "poorer population" or "poorer world" or "developing econom*" or "less developed econom*" or "underdeveloped econom*" or "under developed econom*" or "middle income econom*" or "low income econom*" or "lower income econom*" or "low gdp" or "low gnp" or "low gross domestic" or "low gross national" or "lower gdp" or "lower gnp" or "lower gross domestic" or "lower gross national" or lmic or lmics or "third world" or "lami countr*" or "transitional countr*" or "emerging econom*" or "emerging nation*").mp. [mp=title, abstract, heading word, table of contents, key concepts, original title, tests & measures, mesh word] |
| 15. | 13 and 14 |
| 16. | 4 and 6 and 8 |
| 17. | 14 and 16 |
| 18. | 7 and 17 |
| 19. | limit 18 to (english language and yr="2004 - 2024" and "young adult and adult (19-24 and 19-44)" and english and last 20 years) |
