# Supplementary material for: Barriers and facilitators to sexual and reproductive healthcare access for women with severe mental illness in low- and middle-income countries: A qualitative systematic review and meta-aggregation
Source: Glob Ment Health (Camb). 2026 May 20;13:e119. doi: 10.1017/gmh.2026.10222 (PMC13279974; doi:10.1017/gmh.2026.10222)
Supplement: Chalmeti et al. supplementary material [file S2054425126102222sup001.zip › Supplementary File 3.docx]

| **Synthesized Finding:** | *Limited knowledge of SRH, low risk perception, and poor service availability hinder access to SRH care for women with SMI.* | | | | | |
| --- | --- | --- | --- | --- | --- | --- |
| **Categories** | **B/F** | **Paper** | **Findings** | **Shortened Finding** | **Supporting Evidence** | **Credibility Rating** |
| Beliefs about relationship between mental health care and SRH | B | Bagadia 2020 | Knowledge about the effect of illness during pregnancy was also limited and culturally driven. | Women's knowedge of the effect of SMI on pregnancy is limited. | . . . illness is in my head . . . baby will be in my tummy so nothing will happen . . . baby is the priority. | Unequivocal |
|  |  | Yu 2022 | People felt they lacked medical knowledge and the need for information about pregnancy increased their concerns. | Women feel a lack of medical knowledge surrounding SRH. | “The problem... I think... I got the disease suddenly, I don't know anything about it, no one tells me whether having a child is a problem, I just feel overwhelmed”. | Unequivocal |
|  |  | Rezaie 2020 | Study participants expressed a particular need for information about women's health issues. They identified pregnancy ... and effects of medication on the developing foetus as key topics of concern. Broadly, participants requested more information on the relationship between SMI and feminine identity. | Women demonstrated a need for sexual education concerning SMI and reproductive health. | I do not know if I can get pregnant with my illness. Can I give birth to a healthy baby? I am worried. Child bearing is a woman's greatest wish. | Unequivocal |
| Beliefs about impact of medication on SRH | B | Bagadia 2020 | The beliefs about the effects of medications in pregnancy had not altered despite consultations with a perinatal psychiatrist. | Women's beliefs of negative effects of medication on fertility did not change despite consultation with healthcare professional. | We want a child but because of medications, we are restricted. They (relatives) said she (N) is not getting pregnant because of the medicines. If medicines are stopped then she can have a child. (Mother of 26-year-old N (diagnosed with BPAD)) | Unequivocal |
|  |  | Vijayalakshmi 2024a | Participants believed that their loss of sexual desire was resulted from their mental illness and the medication they were receiving for it. | Women believe that that decrease in sexual desire was a result of psychiatric medication. | “As a patient taking medication, I have a responsibility to look after my health. Sexuality is not as vital as health. Even my partner will not express interest” | Unequivocal |
|  |  | Vijayalakshmi 2024a | Women believed that the symptoms of STIs were caused by the medication they were taking for mental illness. | Women believe that STI symptoms were a result of psychiatric medication. | “I take regular psychiatric drugs. If I do not continue the treatment, I will start to have symptoms. I am not concerned because this white discharge can be related to the medication I am taking” | Unequivocal |
|  |  | Yu 2022 | People with schizophrenia may also have to manage psychiatric symptoms by taking medication during pregnancy. However, medications are hazardous and most participants expressed fear that they may affect their unborn child. | Women believe that psychiatric medication may negatively effect fetus. | I didn't know what to do. Take medicine to treat yourself… But I have heard that some drugs are harmful to babies. I'm concerned about the development of babies if I take drugs during pregnancy. | Unequivocal |
|  |  | Yu 2022 | Some participants said that once pregnant, considering the effect of drugs on the fetus, they may choose to stop taking drugs. Your primary concern is whether discontinuing the medication will lead to a relapse of the disease during pregnancy. | Women stop taking psychiatric medication during pregnancy, but fear relapse of SMI. | In my opinion, no one takes drugs during pregnancy… If I stop taking medicine, I don't know if the disease will recur. | Unequivocal |
|  |  | Rezaie 2020 | Study participants expressed a particular need for information about women's health issues. They identified pregnancy ... and effects of medication on the developing foetus as key topics of concern. Broadly, participants requested more information on the relationship between SMI and feminine identity. | Women reported lack of knowledge of effects of medication on developing fetus | I do not know if I can get pregnant with my illness. Can I give birth to a healthy baby? I am worried. Child bearing is a woman's greatest wish. | Unequivocal |
| Risk-taking behaviors despite knowledge of risks | B | Wainberg 2007 | Even informants knowledgeable about HIV transmission and risk behaviors reported not practicing safer sex: they infrequently used condoms, dental dams, or other methods of preventing HIV transmission (i.e., non-penetrative sex). | Despite being knowledgeable about STIs, women did not practice safer sex. | They (patients) do know about it (prevention); there are people here who are informed about it as well as people who aren’t, the same as with the general population, but in general what makes them not protect themselves is more the search for pleasure …. They do not bother. The family sometimes brings them condoms, but more often than not they don’t even use them. | Unequivocal |
| No perceived need for sex education by women with SMI | B | Raisi 2018 | It means that sex education is not a priority for most of patients with severe mental illnesses. | Women do not think sex education is a priority. | I have not thought about it because I have not had any experience. It has not been an important subject for me. After we experience it, we can better understand it. When we have an experience, we could understand more about. It has not been my concern in acute phase of illness. Hallucinations, emotional pressures, and oppression were the prominent part of my illness, so sexual issues were not important. It will be usually considered as a prominent and important issue just after marriage. | Unequivocal |
|  |  | Raisi 2018 | Some patients said they would have no need for sex education. | Women do not find a need for sex education. | I think that sexual issues are a part of normal human development and I do not feel the need to speak about this issue with physicians | Unequivocal |
| Lack of SRH skills and knowledge amongst mental healthcare professionals | B | Tumwakire 2022 | General lack of knowledge and skills in oﬀering SRH services was expressed by all participants as a major limiting factor. This has stemmed from SRH not being taught during formal training and the continuous medical education (CME) sessions regularly conducted at the hospital. They therefore expressed a great need for training if to be able to add SRH service provision to their routine mental MH care services. | Women report lack of knowledge/skills about SRH amongst mental health professionals. | “I think to me the clinicians may lack the knowledge about those services and they don’t take them as priority.” | Unequivocal |
|  |  | Raisi 2018 | Since most clinicians do not have enough expertize in management and treatment of these problems, they do not talk about. | Healthcare professionals report feeling unable to ask about sexual needs due to inadequate training. | The patients’ sexual needs are completely ignored by the healthcare providers; because we believe that they do not have sexual needs. Patients are prone to risk tak ing sexual behaviors, despite decreased libido due to medications side effects. | Credible |
|  |  | Tumwakire 2022 | All participants acknowledged giving MH issues most priority and almost none to other issues. This was due to the perception that SRH was outside their MH specialty hence didn’t need their attention when attending to patients. | Healthcare professionals believe sexual problems were outside of their specialty. | “For us here in the mental clinic we may not dwell so much on the sexual problem, we usually dwell mostly on the mental problems, hallucinations etc.” | Unequivocal |
| Lack of time for mental healthcare professionals to address SRH | B | Tumwakire 2022 | Participants noted their working environment to be unsupportive for them to oﬀer SRH services in many ways. They reported to have high patient workload making it impossible to spare time for “non-MH issues”. The participants noted that there were no appropriate policies in place to guide them on when and how to add SRH in their routine work | Healthcare professionals said they had insufficient time to offer SRH services, due to heavy workload | “The clinic is usually heavy with very many patients. Unless if the patient raises it specifically that is when you can go into that.” | Unequivocal |
|  |  | Raisi 2018 | It is a reality that symptoms control is superior over the sexual problems (as our participants said) at the time of admission of patient and patients admitted in the acute phase of illness. But in the subsequent session, clinicians must pay attention to sexual problems. This implicitly reflects the problem in mental health care in Iranian society. Psychiatric services are often concentrated around the time of hospitalization and outpatient services are relatively limited. | Healthcare professionals believe SMI symptom control is more important than sexual problems. | Because of limited time, sexual problems are usually ignored in the interviews by the clinicians, especially when they have large number of patients who they need to treat. | Unequivocal |
|  |  |  |  |  |  |  |
|  |  | Raisi 2018 | It is a reality that symptoms control is superior over the sexual problems (as our participants said) at the time of admission of patient and patients admitted in the acute phase of illness. But in the subsequent session, clinicians must pay attention to sexual problems. This implicitly reflects the problem in mental health care in Iranian society. Psychiatric services are often concentrated around the time of hospitalization and outpatient services are relatively limited. | Healthcare professionals believe SMI symptom control is more important than sexual problems. | Duration of admission in acute wards is usually 4–6 weeks and in this period of time, symptoms remission is very important, so sexual health problems are in the second rank. | Unequivocal |
| Lack of policies on how to address SRH in mental healthcare facilities | B | Tumwakire 2022 | The participants noted that there were no appropriate policies in place to guide them on when and how to add SRH in their routine work | Healthcare professionals reported there were no appropriate policies in place to guide them on when and how to add SRH in their routine work | “The clinic is usually heavy with very many patients. Unless if the patient raises it specifically that is when you can go into that.” | Unequivocal |
|  |  | Wainberg 2007 | Staff confirmed that both mental health settings had policies proscribing sexual activity within the hospital. However, how to apply this policy was unclear, staff did not always know how to proceed, and they expressed their confusion. | Healthcare professionals were unaware how to encourage safe sex within psychiatric facilities. | I heard many times, from nurses, nursing assistants and other professionals describing how difficult it was for them and asking me ‘what should we do, if we catch them? Separate them, right?’ It was difficult for them to deal with that. I mean, they knew it was forbidden, of course, but at the moment when things were happening, they didn’t know what to do. To my knowledge, there’s never been a specific training, but only a conversation after some incident. | Unequivocal |
| Lack of knowledge of STI symptoms and treatment amongst women with SMI | B | Vijayalakshmi 2024a | Participants believed that these symptoms were not actually because of any severe illness. It may be because of other associated problems, excessive heat, food allergy, and some belief in themself. | Women believe that STI symptoms were a result of other unrelated health conditions. | “I experience itching and irritation in my perineal area whenever I am in contact with my spouse. My husband smokes, thus I feel that the smoke combined with my blood cause this” | Unequivocal |
|  |  | Vijayalakshmi 2024b | Women were unaware of the symptoms experienced by them. | Women were unaware of the presence of STI symptoms. | Dietary sensitivities are one of the potential causes of this peculiar vaginal discharge. I invariably have a white discharge after eating any heat‑producing food, such as sweets, brinjal, or drumsticks. | Unequivocal |
|  |  |  |  |  | Every time we come into contact; I start to itch | Credible |
|  |  | Vijayalakshmi 2024a | Proper treatment for symptoms of STIs will prevent further damage and promote the reproductive health of WMI. Participants were not having adequate knowledge of the correct treatment options. | Women were unaware of the presence of of STI treatment options. | “I experience itching and white discharge in my perineum, but I have not sought therapy. I believed it to be typical for all women” | Unequivocal |
| Poor knowledge of contraception amongst women with SMI | B | Vijayalakshmi 2024a | Participants were not aware of the temporary and permanent methods of contraception. This may be related to the fact that they knew no method. | Women were unaware of contraceptive methods. | “My husband does not approve of whatever approach I use. As the days passed, I believed my pregnancy would come to an end” | Unequivocal |
|  |  | Vijayalakshmi 2024b | Women claimed they were unaware of the various forms of birth control. | Women were unaware of forms of birth control. | I am uneducated. I consequently lack any approach. There is no one to guide me. Whatever method I employ, my husband does not like it. I thought my pregnancy would terminate as the days went by, | Unequivocal |
| Lack of family planning services | B | Zerihun 2021 | The main issue was that they were not able to obtain family planning services when they needed them. They reported that this was because nobody made an eﬀort to give it to them and they experienced insurmountable barriers to accessing family planning in primary health care. | Women reported a lack of family planning services. | “Health extension workers should teach us and our family. ... They [women with SMI] need to get frequent advice and teaching... Yes, education is good. For a mentally ill women family planning would be good when they give time just like you have given me now and when they ask us and when they help us to understand, until now nobody has done this, this is my first time. | Unequivocal |
| Access to SRH services is wanted by women with SMI | F | Zerihun 2021 | Most of the study participants discussed that family planning services should be accessible for all women living with a mental illness. They spoke about the need for accessibility and privacy, and raised concerns about stigma, lack of adequate knowledge about family planning, and the need for special considerations in the family planning service. Most participants preferred to be provided with family planning services in a mental health clinic and by a mental health professional. | Women expressed a need for family planning services. | We [women with SMI] need extra support, like advising and teaching slowly, as we don’t have faster functioning in understanding lessons/things. But I still insist it is good if mental health physicians could teach us so persistently and with utmost perseverance. | Unequivocal |
|  |  | Zerihun 2021 | Although almost all the participants preferred to receive the service integrated with their mental health care, a few suggested their home as another alternative service area for family planning in women with SMI. The reason for this being that it would reduce the distance they would be required to travel and it would ensure privacy and confidentiality. | Women expressed a need for family planning services incorporated into their SMI care. | Health extension workers should teach us and our family. … They [women with SMI] need to get frequent advice and teaching… Yes, education is good. For a mentally ill women family planning would be good when they give time just like you have given me now and when they ask us and when they help us to understand, until now nobody has done this, this is my first time. | Unequivocal |
|  |  | Tumwakire 2022 | Due to the observed deranged sexuality, all participants noted to recommend FP for all at risk patients. Some of the participants provided room for relatives and patients when stable to make the FP decision ... Participants noted that being on FP was in best interest for the patients and the community to prevent producing children who would not get appropriate care. | Healthcare professionals expressed a need for family planning services for women with SMI. | “Yes, they come here and they involve themselves in sex so eventually it became too much in the compounds and in the meeting we had to decide to administer family planning to them without their consent without their choice. It is now us the health workers basing on the observation we have made to decide what to do for them but not giving them that opportunity.” | Unequivocal |
|  |  | Rezaie 2020 | Study participants also stated that they want to know about the impact of SMI and medication on their sexual relationships. They expressed a desire to have a normal sexual relationship with their husbands. | Women reported a need for sexual education concerning SMI and sexual relations. | As a woman, I must have a sexual relationship with my husband. For some time, my desire has been reduced. I do not know if this is because of the illness or the medications. I wish someone could explain this to me. | Unequivocal |
| General awareness regarding HIV amongst women with SMI | F | Wainberg 2007 | Some patients had basic knowledge about transmission and risk behaviors and reported having learned from governmental public health campaigns, outpatient drop-in groups about sexual health, or because they had been acquainted with patients infected with the virus. | Women have basic knowledge of STIs due to governmental public health campaigns. | …[using condoms] is much more dangerous, the condom might break. | Unequivocal |
| Inclusive and supportive learning environment for women's SRH education | F | Wainberg 2007 | Within the focus groups, patients felt comfortable talking about sex and risk behaviors and discussing sensitive issues; group activities are common to them in their treatment programs. | Women want group sex education interventions. | Well, the first thing that comes to my mind is that this type of work may be very rich if it is done with small groups. | Unequivocal |
|  |  | Wainberg 2007 | Patients and staff also stressed that the intervention needed to use interactive exercises to motivate participants and to allow for ‘hands-on’ learning of information and prevention skills. There were no doubts among participants and staff that Brazilian music, as well as activities with body movement, would need to be woven into the intervention. | Women want interactive exercises to promote comprehensive, tailored SRH education. | I think a theatre play should be created. To teach how to use the female and the male condom. | Unequivocal |
|  |  | Wainberg 2007 | When patients and staff were asked about intervention group membership and facilitators, consensus was clear about having mixed-gender groups run by two facilitators, one female and one male. | Women want mixed-gender interventions for SRH learning environments. | There are two perspectives, the two are different. It brings more discussion. Yet, it would be helpful if there are two facilitators, one of each gender. | Unequivocal |
| Mental healthcare professionals perceive women with SMI to have similar SRH needs to people without SMI | F | Tumwakire 2022 | Participants acknowledged that people with MI have normal sexual feelings which was expressed by patients’ showing interest in health workers, starting relationships and getting married. | Healthcare professionals believe that women with SMI have similar sexual needs to women without SMI. | “We should know that being mentally sick doesn’t take away your sexual feelings, it doesn’t. these are normal women, these are normal men and if they see this young musawo looking nice they will definitely give you sexual advances.” | Unequivocal |
|  |  | Tumwakire 2022 | People with MI engage in relationships and they work towards maintaining the relationship. This desire to maintain relationships is further utilized by health workers as a motivation for treatment adherence to prevent acute episodes that might cause their partners to leave them. | Healthcare professionals believe that relationship maintenance is a big motivator for treatment for women with SMI. | “You normally find that such people they comply to their treatment very well for security of their marriage.” | Unequivocal |
| Condoms viewed negatively | B | Zerihun 2021 | Although condom use was generally recognised as a means of contraception, condom use was more often linked to promiscuity and preventing transmission of sexually transmitted diseases rather than an intervention used in family planning. | Women express negative stigma of promiscuity surrounding condoms. | Condom means… indecent people use condoms; these people use them to create temporary relationships… To protect themselves from diﬀerent problems, when they are in temporary relation. They are ill-mannered. They used it in hotels…. | Unequivocal |
|  |  | Wainberg 2007 | Some staff expressed concerns over the possibility of making condoms available to patients, believing that doing so would encourage sexual activity. The idea that offering condoms to patients could help make condoms more familiar, promote healthy sexuality, and make sex safer also was not expressed by staff. | Healthcare workers believe that access to condoms for women with SMI will encourage sexual activity. | If you give condoms in the ward in a psychiatric hospital like ours and put the guy in there, in the patio (…) so, ‘give me a condom’, cause it’s allowed? What’s he gonna do with the condom? Is he gonna play with it or is he gonna have sex with someone? | Unequivocal |
|  |  | Wainberg 2007 | Other patients reported ‘concerns’ about condoms and their efficacy to prevent transmission, stemming, according to staff and patients, from the position and influence of several Brazilian churches which have taken public positions that condoms are not an effective prevention method. It was not uncommon for patients to ‘believe’ in magical or religious protective mechanisms against getting HIV infection. | Women report misinformation about SRH spread by religious leaders and institutions. | I’ve already met people who got AIDS using condoms that break… …I think that He [God] is aware of everything that He’ll do to me. | Unequivocal |

| **Synthesized Finding:** | *Restricted autonomy and exclusion from health decisions prevent women with SMI from accessing SRH care that reflects their needs and rights.* | | | | | |
| --- | --- | --- | --- | --- | --- | --- |
| **Categories** | **B/F** | **Paper** | **Findings** | **Shortened Finding** | **Supporting Evidence** | **Credibility Rating** |
| Decision-making about SRH decisions done by others | B | Bagadia 2020 | There was a tendency by the subjects to rely upon doctors for decision-making if there were to be a problem related to pregnancy or any adverse effects on the foetus | Women relied on healthcare professionals for decision-making in fetus-related issues. | that is what I don’t know . . .. I’ll come and ask the doctor what to do. We had scanning done, reports are all fine. | Unequivocal |
|  |  | Bagadia 2020 | Majority of the women (88%; n=37) reported reduced autonomy in their decision-making process related to pregnancy. | Women reported decreased autonomy in decision in the pregnancy process. | Not only about child, but I also wasn’t having any feelings about sex, getting involved and doing it; nothing. Just did it as a duty and carried on. | Unequivocal |
|  |  | Bagadia 2020 | In a vital and highly personal decision such as pregnancy and management of mental illness, women relied heavily on their family’s perceptions and seemed to make decisions that would secure their current and future safety within their family. | Women made decisions based on family perceptions and expectations. | to me . . . if you ask me to wait for long it will be difficult I think. If I’m taking tablets . . . if others find out, they will say things like ‘she has an illness’ . . . she’s got some illness so . . .. that’s why she is consulting someone. If one person finds out the whole town will find out. | Unequivocal |
|  |  | Rani 2023 | Another participant expresses how she feels when her partner makes the reproductive decisions without involving her. | Women report feeling helpless when partners making reproductive decisions without consulting them. | I feel so helpless.giving birth to a child is a gift, but it has become a curse for me.and when I was pregnant for the fifth time, he would insist on having sex even a few days before delivery; as a result, I lost my baby and was hospitalized for almost a month. | Unequivocal |
|  |  | Tumwakire 2022 | Due to the observed deranged sexuality, all participants noted to recommend FP for all at risk patients.... some participants just decided for patients. Participants noted that being on FP was in best interest for the patients and the community to prevent producing children who would not get appropriate care. | Healthcare professionals report making family planning decisions for women. | “Yes, they come here and they involve themselves in sex so eventually it became too much in the compounds and in the meeting we had to decide to administer family planning to them without their consent without their choice. It is now us the health workers basing on the observation we have made to decide what to do for them but not giving them that opportunity.” | Unequivocal |
|  |  | Lundberg 2022 | One woman was sexually exploited by the clinician treating her mental illness, who turned a friendly relationship into a sexual relationship when the participant’s family was no longer able, or willing, to pay for the expensive psychiatric medication. ... The economic dependence of this female participant in relationship to her abusive partner made her powerless in decision-making about sex and condom use. | Women report decreased autonomy in sexual decision-making due to economic dependence. | "Sometimes our parents are the ones who put us in such temptations. Because when the man said that he was going to treat me, they should have stopped him and said NO [tapping table], we are going to give you money. But they said thank you musawo [clinician]. Now, doesn’t that mean that you have fully given away your daughter? Yes, you have given away your daughter. Do you think that that man can treat me without anything?" | Unequivocal |
|  |  | Vijayalakshmi 2024a | Health professionals and family members promoted sterilization, indicating that it is the most appropriate method for WMI which may undermine a woman’s autonomy related to fertility-related decisions. | Healthcare professionals and family members push sterilization as the most appropriate form of fertility-related decisions. | “After the birth of my second child, I underwent a tubectomy. My family decided without consulting me. They have agreed not to have children in the future because I take medication, which will have an impact on my health” | Unequivocal |
|  |  | Vijayalakshmi 2024b | WMI has admitted to using various forms of contraception, but they have been unable to do so for multiple reasons, including concerns about their fertility, issues with their current method, and outright refusal to use the technique. Regarding contraception, the decision was mainly resting on their spouse and the in‑laws. | Women report contraception decisions made their partner and in laws. | I had a tubectomy after the birth of my second child. My family chose without my input. I take medication which will affect my health; therefore, they have agreed not to have child in the future. | Unequivocal |
| Contraception avoidance | B | Bagadia 2020 | Irrespective of their ambivalence about pregnancy, most women (79%; n = 33) were sexually active and were not using contraception. They reported that their husbands or extended family members had the most influence on decisions around pregnancy and contraception. | Women are sexually active but not using contraception. | **Dr:** So, at that time you weren’t using any contraception . . . did you have any tension that if you get pregnant what will you do . . . how will you manage? **R:** No tension . . . but there will be a pain, during delivery, isn’t it ? . . . that worried me a little. how will it be ? . . . will I be able to tolerate that pain or not ? . . . I was worried about that . . . that’s all. | Unequivocal |
|  |  | Vijayalakshmi 2024a | Women expressed that there is a refusal to use the method by themselves and by their partners. | Women express a general refusal to use contraception. | “I am having one child, so I believe my family is complete. I am avoiding contact and refraining from employing methods out of concern for my health. My partner also dislikes me using any method” | Unequivocal |
|  |  | Vijayalakshmi 2024b | WMI has admitted to using various forms of contraception, but they have been unable to do so for multiple reasons, including concerns about their fertility, issues with their current method, and outright refusal to use the technique. Regarding contraception, the decision was mainly resting on their spouse and the in‑laws. | Women not using contraception due to concerns about fertility, issues with methods and refusal. | I had a tubectomy after the birth of my second child. My family chose without my input. I take medication which will affect my health; therefore, they have agreed not to have child in the future. | Credible |
| Family makes decisions about sexual abstinence | B | Wainberg 2007 | Another key finding related to content was the important role of family in the lives of psychiatric patients. Many patients live with relatives, some of whom keep close watch on the psychiatric care their loved ones receive. | Women are made to abstain by family members | We have many young adult patients whose relatives do anything they can to prevent their children from having sex, and I mean anything! | Unequivocal |
| Mental healthcare workers fearful to intervene in inpatient sexual violence cases | B | Rani 2023 | Sexual exploitation occurs when a perpetrator takes advantage of participants’ vulnerable and dependent states by forcing them to witness and participate in sexual acts. | Healthcare professionals refrain from intervening in sexual violence to protect victims who fear retaliation. | Sexual violence almost becomes the cost a person must pay for the care they get from family or relatives. I do come across many of these problems. Often what limits me from taking any action is that the victim does not want any action to be taken as they fear consequences in the form of abandonment or retributions. | Unequivocal |
|  |  | Tumwakire 2022 | The patient- health workers is also significantly aﬀected due to their aﬀected sexuality. Some of the participants expressed fear due to the sexual assault experiences in the hospital. They also noted aggressiveness among patients especially if they try to interfere with their sexual activities. This aﬀected their work with some participants noting scenarios where they had to avoid providing care to some of the patients. | Healthcare professionals express fear of being sexually assaulted by women with SMI, leading to avoiding providing care to some patients. | “Female patients develop feelings and they even brand you names because they like you and you end up feeling uncomfortable.” | Unequivocal |
| Women desire spousal support in SRH issues | F | Yu 2022 | Regarding childbirth, spouses can provide emotional support to participants in several ways. On the one hand, participants expected that their spouse could confront fertility-related issues with them and could compensate for their insufficient ability to care for children. | Women want to involve partner in fertility-related SRH issues. | “Maybe, I would like my husband to support me, mentally, help me, maybe let off steam sometimes. I wish we could bear these risks of reproduction together”. | Unequivocal |
| Positive attitude towards contraception | F | Zerihun 2021 | Despite this they reported that contraception was important in preventing pregnancy and expressed a positive attitude towards its use. | Women have positive attitudes towards contraception. | For me, a woman living with mental illness shall use implant earlier or, if she wants to have sex, she shall use pills or injection so that she can prevent extra mental health complications associated with such issues. | Unequivocal |

| **Synthesized Finding:** | *Stigma around mental illness and sexuality, reinforced by societal norms, discourages care-seeking for the SRH needs of women with SMI.* | | | | | |
| --- | --- | --- | --- | --- | --- | --- |
| **Categories** | **B/F** | **Paper** | **Findings** | **Shortened Finding** | **Supporting Evidence** | **Credibility Rating** |
| Stigma around mental illness | B | Bagadia 2020 | For the women who had some support from family in making decisions, stigma and concerns about the behaviour of the family members towards them contributed largely to their thought process | Familial treatment influences women's thought process concerning SRH care. | **Dr:** So, at that time you weren’t using any contraception . . . did you have any tension that if you get pregnant what will you do . . . how will you manage? **R:** No tension . . . but there will be a pain, during delivery, isn’t it ? . . . that worried me a little. how will it be ? . . . will I be able to tolerate that pain or not ? . . . I was worried about that . . . that’s all. | Credible |
| Gender preference for baby | B | Bagadia 2020 | In some instances, the desire for a male baby meant a rapid succession of pregnancies for the mother. | Women's desire for a male child leads to delayed sterilization and abortion of female fetus. | . . . one more pregnancy and if I have a boy, we’ll get operated (tubal ligation). The first time, a girl was born but she died. Elder daughter is there. The second time, I had one, that was daughter again and this is what happened to her. Then, the third one was the abortion I had done. Then one more I lost at 5 months. So, this way I’ve lost three till now . . . | Unequivocal |
| Mental healthcare professionals feel uncomfortable discussing SRH | B | Raisi 2018 | Sexual issues are culture-based and it is not easy for individual to speak about it. | Healthcare professionals report feeling unable to ask about sexual needs due to culture. | In our community, talking about sexual issues is very difficult for clinicians. How could a clinician educate patients regarding sexual issues when he/she does not feel comfortable in talking about these concerns? In psychotherapy, the patient does not speak about a subject that is difficult for the therapist. Clinicians should ask some questions about sexual concerns for helping their patients; however, they are uncomfortable with these issues. | Unequivocal |
| Women with SMI feel shame when talking about SRH | B | Raisi 2018 | One of the important reasons for not talking about sexual problems is shame. The related codes are “not feeling comfortable about sexual problem,” “shame due to lack of information, “and “not speaking about sexual concern, as a value.” | Women feel shame in talking about sexual problems. | These problems are hidden topics and mostly, patients talk about them after a few sessions. I always ask questions about sexual issues with caution; group of patients respond, but some of them prefer to answer in a private setting (not in front of their relatives even their spouse). Then, they might say that they could not have sexual relations and ask for help. This is a common scenario and the patient is usually sad. | Unequivocal |
|  |  | Zerihun 2021 | Despite her concerns, this woman had never raised this issue for discussion with a health professional or others, and no one had given her any information about this issue. | Women are not comfortable discussing SRH with healthcare professionals. | She [a woman with mental illness] is on psychiatric medication and if she gets pregnant and gives birth, what is going to happen to the newborn, is he going to be mentally retarded or normal? I only ask myself about this, I never ask or talk with the health workers or with others. | Unequivocal |
|  |  | Raisi 2018 | Although in some cases, sexual problem to be mentioned indirectly by patients and a group of patients pointed to their sexual problems indirectly. | Sexual problems are not raised by women due to cultural norm of pre-marital abstinence. | Sexual relations are allowable only after marriage in our culture, so they (patients) always ask questions about when they get married; some of the patients’ behaviors are a symbolic reaction to their sexual needs. I think this is very important, especially in psychotic patients | Unequivocal |
| Concern about disclosure | B | Raisi 2018 | Sometimes patients are worried about the probable annoying content of clinician's comments which could originate from two main factors including: hearing the reality of their sexual problems, improper performance of clinician and this reality in context of Iran “that sexual relations are socially no acceptable unless after marriage.” | Women are worried about private SRH information being unduly disclosed to others by healthcare professionals. | My doctor told him (my fiancé) that he must be my caregiver for the whole of his life. He (The clinician) believed that I could not be a good mother. My fiancée wasn't worried before that. We had had a good relationship… after the session; he was afraid and really disappointed. | Unequivocal |
| Women with SMI are perceived to be incapable of raising children | B | Tumwakire 2022 | This negative eﬀect on relationships was also noted to occur with their children with majority having others looking after the children due to their inability to provide care. | Healthcare professionals believe that women with SMI have decreased caretaking abilities. | “They have to continue with it because some of them might have other babies, yet they don’t have the money to look after them. | Unequivocal |
|  |  | Tumwakire 2022 | This inability to look after children by people with MI was one of the reasons given by both male and female participants for giving FP services to most patients. | Healthcare professionals report administering family planning services to women with SMI due to perceived inability to take care of children. | “After they have delivered and we have failed to identify their family, we take care of the children after delivery or we give them to the baby care centers like watoto especially if the patient is unable to stay with those children.” | Unequivocal |
|  |  | Zerihun 2021 | All the participants reported that most family members, the community and some health professionals were of the view that a woman with mental illness should not have children. | Women with SMI believe that others do not think that they should have children. | ……. How can she get pregnant if the illness doesn’t disappear? God’s work… People talk, saying why she didn’t get contraceptive injections and why she wanted to have children since she is ill… but pregnancy can come against her will by force… all people say no giving birth if she is mentally sick. | Unequivocal |
| Gendered family roles prioritized over SRH concerns | B | Vijayalakshmi 2024a | Women played various other roles in the family, like mother, wife, sister, and so on. They often feel that fulfilling these roles is more important than sexual desire. | Women believe that prescribed gender roles are more important than sexual desire. | “My brothers and children will be in my care. They are becoming older; therefore, I need to focus on their education and development rather than my sexual desires” | Unequivocal |
|  |  | Vijayalakshmi 2024a | As a result of family issues, women are paying less attention and are not concerned about the symptoms of STIs. | Women are not concerned about STI symptoms due to family issues. | “My vaginal discharge has lasted for three months. I am unable to focus on this issue because of other problems in my family (my son’s love marriage, my husband’s drinking and my in-laws’ health concerns)” | Unequivocal |
|  |  | Vijayalakshmi 2024b | WMI explored that they were not given attention to the STI symptoms because of family issues and treatment adherence to mental illness. | Women ignore STI symptoms due to family issues. | I had vaginal discharge for three months. Due to other family issues, I cannot concentrate on this. | Unequivocal |
| Mental healthcare professionals assume sexual behaviors are due to SMI | B | Wainberg 2007 | Some staff viewed their patients’ sexual activity and lack of consistent condom use in terms of pathology as opposed to normative behavior. | Healthcare professionals view women's sexual habits as being attributable to their illness. | Sometimes I think that psychiatric patients, depending on our attitudes, also lack the capacity to be reasonable, their personality, I don’t know if we can call it that, but they suffer from some symptoms, you know? | Unequivocal |
| Misunderstanding around family planning services | B | Zerihun 2021 | From most of the participants, there was initial resistance to talk about their knowledge of family planning which appeared to be related to the sensitivity of the topic. Most of them equated family planning with prevention of birth, rather than planned birth, and referred only to contraceptive interventions. | Women associated family planning with prevention of birth rather than planned birth | I think it [Family Planning] is a business. …. Business is going to males to get money…. Women who do that, they know well about it because they are afraid to get pregnant. | Unequivocal |
| Opportunities for peer education on SRH valued by women with SMI | F | Wainberg 2007 | In addition to protecting themselves, patients also expressed their wish and need to learn more about HIV prevention so that they could share that knowledge with relatives, friends, and fellow patients; they valued their community membership and expressed their sense of “social responsibility.” | Women express a desire to learn more about STI prevention in order to educate community. | It could be of great utility, I could even pass what I will learn to others. | Unequivocal |
| Sex-positive attitudes and environment | F | Wainberg 2007 | Numerous field observations notes reported patient couples holding hands. The clinic/hospital grounds were an important place in patients’ social lives, and romantic and/or sexual encounters took place within the confines of the psychiatric clinics they regularly attended. | Women feel more comfortable pursuing romantic and sexual relationships within SMI treatment facilities. | I think it is, undoubtedly, easier for [patients] to… meet other partners here. Partially because this is the place they often come to. Cause where do we end up meeting boyfriends? Where we usually are, whether at the workplace, or in college, or in our group of friends. | Unequivocal |
|  |  | Wainberg 2007 | Throughout all phases of the ethnography, it was evident that psychiatric patients felt comfortable talking about sex. The ethnographic observers found no reservation among patients when the goals of the study were presented to them; quite the opposite, the ethnographers were sought out by the patients, a phenomenon documented in innumerable field notes. | Women have sex-positive attitudes and are comfortable talking about SRH. | After I responded what type of research we are conducting, several patients asked me to be their psychologist so they could talk about sex with a mental health professional. | Unequivocal |
| Perceived rewards of motherhood | F | Yu 2022 | Maternity is one of the most crucial roles women play in their lives. The participants felt that becoming a mother would allow them to live a happier life. It makes participants feel‘normal’ to raise children. | Motherhood is a positive reinforcement for women with SMI. | “In my opinion, people should have their own children in the world, so their life is relatively complete. I also want to have a child to call me mom and then raise her to grow (…) and let me feel like I'm the same as normal people”. | Unequivocal |
|  |  | Yu 2022 | When they are informed and optimistically seek solutions, they worry about reducing fertility, where the power of a positive coping style is reflected. | Women with SMI exhibit positive attitudes towards SRH care. | “I said to myself that don't be afraid, because medical technology is so developed. I can go to the hospital to check the baby's health condition when I have a child”. | Unequivocal |
|  |  | Yu 2022 | Motherhood makes them feel needed and has a strong sense of responsibility, making them more active in treatment and life. | Motherhood encourages women to seek psychiatric treatment. | “I'm tired of living, I don't want to live a few times (before)....now I'd try to endure it because I cannot let my daughter have no mother, how unhappy a child is without her mother”. | Unequivocal |
