# Supplementary material for: Barriers and facilitators to sexual and reproductive healthcare access for women with severe mental illness in low- and middle-income countries: A qualitative systematic review and meta-aggregation
Source: Glob Ment Health (Camb). 2026 May 20;13:e119. doi: 10.1017/gmh.2026.10222 (PMC13279974; doi:10.1017/gmh.2026.10222)
Supplement: Peer review history

# Reviewer report: Review: Barriers and facilitators to sexual and reproductive healthcare access for women with severe mental illness in low- and middle-income countries: A qualitative systematic review and meta-aggregation — R0/PR2

**DOI:** 10.1017/gmh.2026.10222.pr2

This manuscript offers a well-structured and timely qualitative synthesis examining barriers and facilitators to sexual and reproductive health (SRH) care for women with severe mental illness (SMI) in low- and middle-income countries (LMICs). The topic is highly relevant to global mental health, aligns with current international policy priorities, and addresses a population that is consistently underrepresented in research and service-planning discussions. The authors follow the Joanna Briggs Institute (JBI) meta-aggregation approach and report the review in accordance with PRISMA guidelines. The manuscript is logically organized, clearly written, and the main arguments are presented in a coherent and accessible way.

The review succeeds in bringing together findings from diverse contexts and in highlighting cross-cutting themes around knowledge gaps, service availability, autonomy, and stigma. The synthesized findings demonstrate consistency and are supported by appropriate data, and the discussion relates these findings to broader theoretical models and existing literature.

There are, however, several areas where the manuscript could be strengthened to improve clarity, methodological transparency, and conceptual nuance.

1. Scope and Framing

The introduction establishes a strong rationale, but the conceptual framing could be sharpened. The manuscript references the Levesque et al. five-dimension model of access, yet the integration of this framework into the results and discussion is uneven. Strengthening the explicit links between synthesized findings and these dimensions—perhaps in a dedicated subsection—would improve conceptual clarity.

Similarly, the definition of “severe mental illness” could benefit from more precision. The inclusion criteria specify diagnoses such as schizophrenia, bipolar disorder, and severe depression, but the boundaries of “severe depression” vary across settings and may introduce heterogeneity into the sample.

2. Methods and Transparency

The methodological description is generally thorough, but several clarifications would enhance robustness:

• It is noted that 30% of titles and abstracts were double-screened. A brief rationale for this limited proportion, and a comment on how discrepancies were resolved, would improve transparency.

• More detail is needed on how credibility ratings (unequivocal vs. credible) were applied to extracted findings.

• Although the authors state that unsupported findings were removed before synthesis, the rationale for their removal—rather than downgrading—could be elaborated, as this choice may affect the breadth of included perspectives.

3. Interpretation of Findings

The findings are presented clearly, but at times the interpretations may over-generalize from limited data:

• The manuscript notes the lack of exploration of alternative care-seeking pathways (e.g., religious or traditional healers), but also references related evidence from other domains. A more cautious approach is advisable to avoid overstating conclusions beyond the included data.

• Recommendations regarding digital health solutions and integration of services are reasonable, yet the link between these recommendations and data from the included studies is somewhat indirect.

4. Contextual Considerations

Given that the synthesis draws from only six countries, the manuscript should more explicitly acknowledge the limits of cross-context comparability. Several studies come from tertiary or specialized psychiatric settings, which may not reflect broader realities of SRH access in LMICs.

Additionally, while the review highlights the influence of stigma, cultural norms, and gendered expectations, the manuscript could better differentiate between barriers that disproportionately affect women with SMI and those that affect women in LMICs more generally.

Minor Comments

• Terminology: In the synthesized findings section, there is a repeated use of “(c)” in the results’ listing; this should be corrected to maintain continuity.

• Figures: Figures 2–5 are helpful but could benefit from brief explanatory captions summarizing their structure for readers.

• Typographical issues: A few minor typographical inconsistencies appear across the manuscript (e.g., spacing, punctuation), which can be addressed during copyediting.

• Supplementary materials: The manuscript frequently references supplementary files; ensuring these are complete and well-cross-referenced will be important at final submission.

# Reviewer report: Review: Barriers and facilitators to sexual and reproductive healthcare access for women with severe mental illness in low- and middle-income countries: A qualitative systematic review and meta-aggregation — R0/PR3

**DOI:** 10.1017/gmh.2026.10222.pr3

Thank you for the opportunity to review the submission.

I would like to congratulate the authors for this work on an important and relevant topic on women’s sexual and reproductive health (SRH). Here are my suggestions for their kind considerations:

Introduction

May I suggest starting with the definition of SRH & then raising the issue of access?

There are few considerable & strong statements without any references that needs attention.

To make this section succinct, easier to read and better flow, I suggest a separate paragraphs on SRH care among women in LMICs followed by women with severe mental illness (SMI). It will be important to highlight and argue why women with SMI in LMICs needs distinct research with inclusion of gender disadvantage and sociocultural determinants in these settings. This may be supplemented by a critical scrutiny of earlier systematic reviews (Brown et al 2025& Sisodia et al 2025) and HIC focussed review of SRH among women, to strengthen the rationale for this review.

I am wondering about the position and relevance of paragraph on the five dimensions of health care accessibility in the current form. It might be helpful to reframe and include in the first paragraph on SRH/access or in Methods?

Methods

Overall well- written section with few suggestions/comments for consideration:

It is mentioned that studies focussed on women with depression, postpartum depression and anxiety disorders are excluded but SMI does include severe depression. This exclusion needs clarification.

Study selection years 2004-2024 for relevance to current health system is ok but given the small number of included studies and from limited countries, is it possible to expand the search beyond these years. Earlier research if completed in this domain may be informative and useful for recommendations.

My major concern is lack of double screening. It is unclear how the full text & finals election was completed. Lack of double screening raises serious concerns about the validity of the results and contrary to the standardised systematic review protocol. A comment about process to resolve differences among the authors, if double screening was completed. I note the limitation in screening was acknowledged in the limitations but unclear if screening to final selection was done by a single author only with a need for explanation.

Results

Well- written section with the following minor suggestions for consideration:

Table 1- It might be more readable by categorization by women/caregivers/health professionals or methodology or country/year. The rationale for current description is unclear to me.

It will be helpful to provide a summary of group findings before describing individual study results on different Synthesized Findings.

The write up refers to “Studies” but provides only one reference at various parts of this section. Please do the needful.

Discussion

Ref at the end of the first paragraph is confusing. I understand you are providing reference to five dimensions but likely not needed if already provided in the Introduction and the paragraph is about the finding of this review.

Overall the section is well written but may need to provide broader context with the inclusion of gender disadvantages and sociocultural norms/diversity and sensitivity in various LMICs settings. This section can be further enriched by incorporating findings from HIC settings and highlighting the differences and need for nuanced approach in LMICs. For example, age 17 refers to advantage of community health workers in their intimate knowledge but these can be a significant barrier in these settings.

Intersectorial approach has been mentioned but may need more emphasis in the section given the contribution of multiple factors related to gender, societal, cultural & health care systems especially for women in general and with SMI in LMICs. For example: role of non-health sector

Recommendations mentions at policy levels but these are mostly seem to driven practices. A different section on policies and practices can be considered, For example- gender empowerment measures

# Editor report: Recommendation: Barriers and facilitators to sexual and reproductive healthcare access for women with severe mental illness in low- and middle-income countries: A qualitative systematic review and meta-aggregation — R0/PR4

**DOI:** 10.1017/gmh.2026.10222.pr4

**Affiliations:** School of Public Health and Preventive Medicine, Monash University Faculty of Medicine Nursing and Health Sciences, Australia

Please address all the revisions suggested by the reviewers.

# Editor report: Decision: Barriers and facilitators to sexual and reproductive healthcare access for women with severe mental illness in low- and middle-income countries: A qualitative systematic review and meta-aggregation — R0/PR5

**DOI:** 10.1017/gmh.2026.10222.pr5

**Affiliations:** London School of Hygiene & Tropical Medicine, United Kingdom of Great Britain and Northern Ireland

No accompanying comment.

# Editor report: Recommendation: Barriers and facilitators to sexual and reproductive healthcare access for women with severe mental illness in low- and middle-income countries: A qualitative systematic review and meta-aggregation — R1/PR7

**DOI:** 10.1017/gmh.2026.10222.pr7

**Affiliations:** School of Public Health and Preventive Medicine, Monash University Faculty of Medicine Nursing and Health Sciences, Australia

Thank you for submitting the revised paper, which I am pleased to accept.

# Editor report: Decision: Barriers and facilitators to sexual and reproductive healthcare access for women with severe mental illness in low- and middle-income countries: A qualitative systematic review and meta-aggregation — R1/PR8

**DOI:** 10.1017/gmh.2026.10222.pr8

**Affiliations:** London School of Hygiene & Tropical Medicine, United Kingdom of Great Britain and Northern Ireland

No accompanying comment.
